# Quantifying Absolute Neutralization Titers against SARS-CoV-2 by a Standardized Virus Neutralization Assay Allows for Cross-Cohort Comparisons of COVID-19 Sera

**DOI:** 10.1128/mBio.02492-20

**Published:** 2021-02-16

**Authors:** Kasopefoluwa Y. Oguntuyo, Christian S. Stevens, Chuan Tien Hung, Satoshi Ikegame, Joshua A. Acklin, Shreyas S. Kowdle, Jillian C. Carmichael, Hsin-Ping Chiu, Kristopher D. Azarm, Griffin D. Haas, Fatima Amanat, Jéromine Klingler, Ian Baine, Suzanne Arinsburg, Juan C. Bandres, Mohammed N. A. Siddiquey, Robert M. Schilke, Matthew D. Woolard, Hongbo Zhang, COVIDAR Argentina Consortium, Andrew J. Duty, Thomas A. Kraus, Thomas M. Moran, Domenico Tortorella, Jean K. Lim, Andrea V. Gamarnik, Catarina E. Hioe, Susan Zolla-Pazner, Stanimir S. Ivanov, Jeremy P. Kamil, Florian Krammer, Benhur Lee

**Affiliations:** a Department of Microbiology, Icahn School of Medicine at Mount Sinai, New York, New York, USA; b Division of Infectious Diseases, Department of Medicine, Icahn School of Medicine at Mount Sinai, New York, New York, USA; c James J. Peters VA Medical Center, Bronx, New York, USA; d Department of Pathology, Molecular, and Cell-Based Medicine, Icahn School of Medicine at Mount Sinai, New York, New York, USA; e Department of Microbiology and Immunology, Louisiana State University Health Science Center Shreveport, Shreveport, Louisiana, USA; f COVIDAR Argentina Consortium, Buenos Aires, Argentina; The Peter Doherty Institute for Infection and Immunity

**Keywords:** COVID-19, SARS-CoV-2, convalescent-phase plasma, neutralizing antibodies, viral neutralization assay

## Abstract

The global coronavirus disease 2019 (COVID-19) pandemic has mobilized efforts to develop vaccines and antibody-based therapeutics, including convalescent-phase plasma therapy, that inhibit viral entry by inducing or transferring neutralizing antibodies (nAbs) against the severe acute respiratory syndrome coronavirus 2 (SARS-CoV-2) spike glycoprotein (CoV2-S). However, rigorous efficacy testing requires extensive screening with live virus under onerous biosafety level 3 (BSL3) conditions, which limits high-throughput screening of patient and vaccine sera. Myriad BSL2-compatible surrogate virus neutralization assays (VNAs) have been developed to overcome this barrier. Yet, there is marked variability between VNAs and how their results are presented, making intergroup comparisons difficult. To address these limitations, we developed a standardized VNA using CoV2-S pseudotyped particles (CoV2pp) based on vesicular stomatitis virus bearing the *Renilla* luciferase gene in place of its G glycoprotein (VSVΔG); this assay can be robustly produced at scale and generate accurate neutralizing titers within 18 h postinfection. Our standardized CoV2pp VNA showed a strong positive correlation with CoV2-S enzyme-linked immunosorbent assay (ELISA) results and live-virus neutralizations in confirmed convalescent-patient sera. Three independent groups subsequently validated our standardized CoV2pp VNA (*n* > 120). Our data (i) show that absolute 50% inhibitory concentration (absIC_50_), absIC_80_, and absIC_90_ values can be legitimately compared across diverse cohorts, (ii) highlight the substantial but consistent variability in neutralization potency across these cohorts, and (iii) support the use of the absIC_80_ as a more meaningful metric for assessing the neutralization potency of a vaccine or convalescent-phase sera. Lastly, we used our CoV2pp in a screen to identify ultrapermissive 293T clones that stably express ACE2 or ACE2 plus TMPRSS2. When these are used in combination with our CoV2pp, we can produce CoV2pp sufficient for 150,000 standardized VNAs/week.

## INTRODUCTION

Severe acute respiratory syndrome coronavirus 2 (SARS-CoV-2) is an enveloped, positive-sense, single-stranded RNA (+ssRNA) virus from the family *Coronaviridae*. SARS-CoV-2 is related to, but not derived from, SARS-CoV, which we will refer to as SARS-CoV-1 for clarity. SARS-CoV-1 and SARS-CoV-2 belong to the genus *Betacoronavirus* and group together as sarbecoviruses, a subgenus that also contains numerous bat “SARS-like” CoVs (1). SARS-CoV-1 caused a limited epidemic of SARS from 2002 to 2004, infecting ∼8,000 people and killing 774 (2, 3). SARS-CoV-1 was ultimately contained and has not reappeared. SARS-CoV-2 is the causative agent for coronavirus disease 2019 (COVID-19). The Chinese government first reported a cluster of 40 cases of atypical pneumonia (now known to be COVID-19) to the WHO on 30 December 2019. Since then, SARS-CoV-2 has erupted into a global pandemic, resulting in tens of millions of cases and more than a million deaths (4).

The emergence and spread of SARS-CoV-2 have required a global response to mitigate the fallout from the pandemic. As a result, the highest priorities for governments around the world are prevention, treatment, and monitoring of infection and immunity (5). Understanding and monitoring immune responses to SARS-CoV-2 are critical for the development of antibody-based therapeutics and vaccines. Both are challenging to study at the necessary scale due to the inherent danger of working with live virus and because of limited access to high-level biosafety containment facilities (i.e., biosafety level 3 [BSL3]). However, the development of pseudotyped viral particles capable of recapitulating SARS-CoV-2 entry—without the dangers or limitations of working with live virus—addresses these concerns. Many such pseudotype virus (PsV) systems based on a lentivirus or vesicular stomatitis virus (VSV) backbone have been published (6–9). These PsV systems have been used to understand and assess humoral immunity in acute-phase and recovered COVID-19 patients and to screen for therapeutic entry inhibitors, such as small molecules, monoclonal antibodies, or convalescent-phase sera. Most importantly, such a surrogate BSL2 virus neutralization assay (VNA) is needed to screen for vaccine-induced responses in domestic animals and humans as the world rushes to develop candidate vaccines against SARS-CoV2.

As of this writing, at least five SARS-CoV-2 vaccine developers have reported phase I/II results involving over 1,700 participants (10–15). While each group claims promising results, it is difficult to compare vaccine-induced immune responses between the various vaccine platforms. This is not only due to a lack of a standardized reporting but also due to a lack of standardized assays for reporting virus neutralization titers. Furthermore, at least 16 studies have reported 350 patients receiving convalescent-phase plasma therapy for COVID-19. Across all 16 plasma studies, some groups establish enzyme-linked immunosorbent assays (ELISAs) or live-virus neutralization thresholds to screen donor plasma, while others do not report binding or neutralization data (16–33). Notably, none of these studies report using a PsV VNA to screen donor plasma. These discrepancies in screening methods/metrics limit the ability to compare across groups and make it difficult to draw conclusions about the quality/potency of antibody transferred to the recipient (32, 33).

A standardized VNA that provides robust, high-throughput results (>100,000 infections/week), generates absolute virus neutralization titers (VNT), and is easily “kit-able” due to its repeatability and ease of use would allow for meaningful comparisons across different labs. In addition to helping down-select the myriad vaccine candidates, use of a standardized VNA to report VNT in absolute units can crowd-source the immense effort being expended by multiple labs across the globe to better understand the basis of the marked variation in VNT seen in recovered COVID-19 patients (34, 35).

The SARS-CoV-2 spike glycoprotein (S) is embedded in the viral envelope and facilitates both receptor recognition and membrane fusion. SARS-CoV-2-S is 1,273 amino acids in length and, as in other coronaviruses, is a trimeric class I fusion protein (36). The S glycoprotein contains two subunits, the N-terminal S1 subunit and the C-terminal S2 subunit. The S1 subunit contains the receptor-binding domain (RBD), which is responsible for host receptor binding. The S2 subunit contains the transmembrane domain, cytoplasmic tail, and machinery necessary for fusion, notably the fusion peptide and heptad repeats (37, 38). Angiotensin-converting enzyme 2 (ACE2), a cell surface enzyme in a variety of tissues, facilitates the binding and entry of SARS-CoV-2 (39–41). However, ACE2 alone is not sufficient for efficient entry into cells. While entry depends on the S1 subunit binding ACE2, entry is further enhanced by proteolytic cleavage between the S1/S2 and S2′ subunits. For both SARS-CoV-1 and SARS-CoV-2, this cleavage-mediated activation of S-mediated entry is supported by the expression of cell-associated proteases, like cathepsins or transmembrane protease serine 2 (TMPRSS2), or the addition of exogenous proteases that mimic the various trypsin-like proteases present in the extracellular lung milieu (39, 42–51). These proteases facilitate entry at the cell surface or via an endosomal route in a cell type-dependent manner. Extracellular proteases are thought to play a pathophysiogical role in the lung tissue damage caused by unabated Middle East respiratory syndrome CoV (MERS-CoV), SARS-CoV-1, and likely SARS-CoV-2 replication (49, 50). Thus, in order to represent SARS-CoV-2 cell entry faithfully, a VNA must be sensitive not only to ACE2 binding but also to the proteolytic activation of spike.

In addition to having a role in receptor binding and entry, S is the primary surface glycoprotein and is the major target of the neutralizing antibody response (52–56). Patients infected with SARS-CoV-2 typically seroconvert within 2 weeks of symptom onset, with about half developing antibodies within 7 days (57–59). Antibody titers appear to be durable at greater than 40 days postinfection (58), but in the case of SARS-CoV-1, reductions in IgG-positive titers begin around 4 to 5 months postinfection and show a significant drop by 36 months (60). Although there are reports of SARS-CoV-2-infected individuals testing positive by reverse transcription-PCR (RT-PCR) weeks after being confirmed as recovered by two consecutive negative tests, these are more likely the result of false-negative results than of reinfection (61, 62). Multiple groups have shown that fully recovered rhesus macaques previously infected with SARS-CoV-2 are refractory to reinfection, at least within 4 weeks of the primary challenge (63, 64). However, a better understanding of the durability and efficacy of the neutralizing antibody response in patients previously infected with SARS-CoV-2 is of paramount importance. Not only do IgG titers wane in the case of SARS-CoV-1, but reinfection is possible in other endemic human coronaviruses (HCoVs), such as 229E, NL63, and OC43, in as little as a year (65–67). Whether the waning of neutralizing SARS-CoV-2 antibodies impacts susceptibility to reinfection is an urgent question that needs to be answered by longitudinal follow-up studies (68–71).

Humoral immune responses to the SARS-CoV-2-S protein are typically evaluated by an ELISA and its many variants (chemiluminescence immunoassay [CLIA], lateral-flow assay [LFA], etc.). These serological binding assays rightfully play a central role in determining patient antibody responses and can complement diagnostics and sero-epidemiological studies, especially when combined with antibody subclass determination (IgM, IgA, and IgG) (72–74). Nonetheless, as many antibodies generated to the spike protein bind but do not block virus entry (75–78), ELISA-based assays that detect titers of spike-binding antibodies cannot always correlate perfectly with neutralizing antibody titers as measured by plaque reduction neutralization or microneutralization (MN) tests (74, 79–82). Even a cleverly designed competitive ELISA set up to detect antibodies that block the binding of the RBD to ACE2 (76, 83) cannot capture the universe of neutralizing antibodies targeted to a conformationally dynamic trimeric spike on a virion (84, 85). The gold standard for detecting antiviral antibodies remains the virus neutralization assay. Assays that faithfully recapitulate entry of SARS-CoV-2 while maximizing safety, speed, and scalability will be vital in the coming months and years. They will enable monitoring of a patient’s neutralizing antibody response, the efficacies of vaccines and entry inhibitors, and the screening of convalescent-phase plasma from recovered COVID-19 patients (57, 86).

In order to meet this need while maximizing safety, speed, and scalability, we generated SARS-CoV-2 pseudotyped viral particles (CoV2pp) by using vesicular stomatitis virus bearing the *Renilla* luciferase gene in place of its G glycoprotein (VSVΔG-rLuc). This approach has been used safely by our group and others to study viruses that would otherwise require significant biosafety constraints, including Ebola virus, Nipah virus (NiV), and, most recently, SARS-CoV-2 (6, 8, 87–90). Here, we present a detailed protocol for the production of CoV2pp, characterize the contributions of stable expression of ACE2 as well as endogenous or exogenous proteases on entry, and standardize the production and performance characteristics of these CoV2pp for use in a robust high-throughput VNA. We have sent out our standardized CoV2pp as ready-to-use “out-of-the-box” VNAs, handling ≥1,000 infections/request, to multiple labs across three continents. We show here the validation of our CoV2pp in a standardized VNA by four independent groups spread across two continents using serum samples from geographically distinct and ethnically diverse cohorts. Lastly, we utilized our standardized CoV2pp and VSV-Gpp in a screen to identify two ultrapermissive 293T cell clones that stably express either ACE2 alone or ACE2 and TMPRSS2. These isogenic cell lines support SARS-CoV-2 entry via endocytic trafficking (i.e., 293T-ACE2 cells for the “late” pathway) or via fusion at or near the cell surface (i.e., 293T-ACE2/TMPRSS2 for the “early” pathway) (40, 45, 50, 91–93). These ultrapermissive 293T clones allow for use of unpurified virus supernatant from our standard virus production batch, which can now provide for ∼150,000 infections per week (96-well format) with no further scale-up. In sum, we have generated a standardized, scalable, high-throughput BSL2-compatible CoV2pp VNA that can provide robust metrics (absolute 50% inhibitory concentration [absIC_50_], absIC_80_, absIC_90_) for meaningful comparisons between labs.

## RESULTS

### Production of VSVΔG-rLuc bearing SARS-CoV-2 spike glycoprotein.

**FIG 1:**
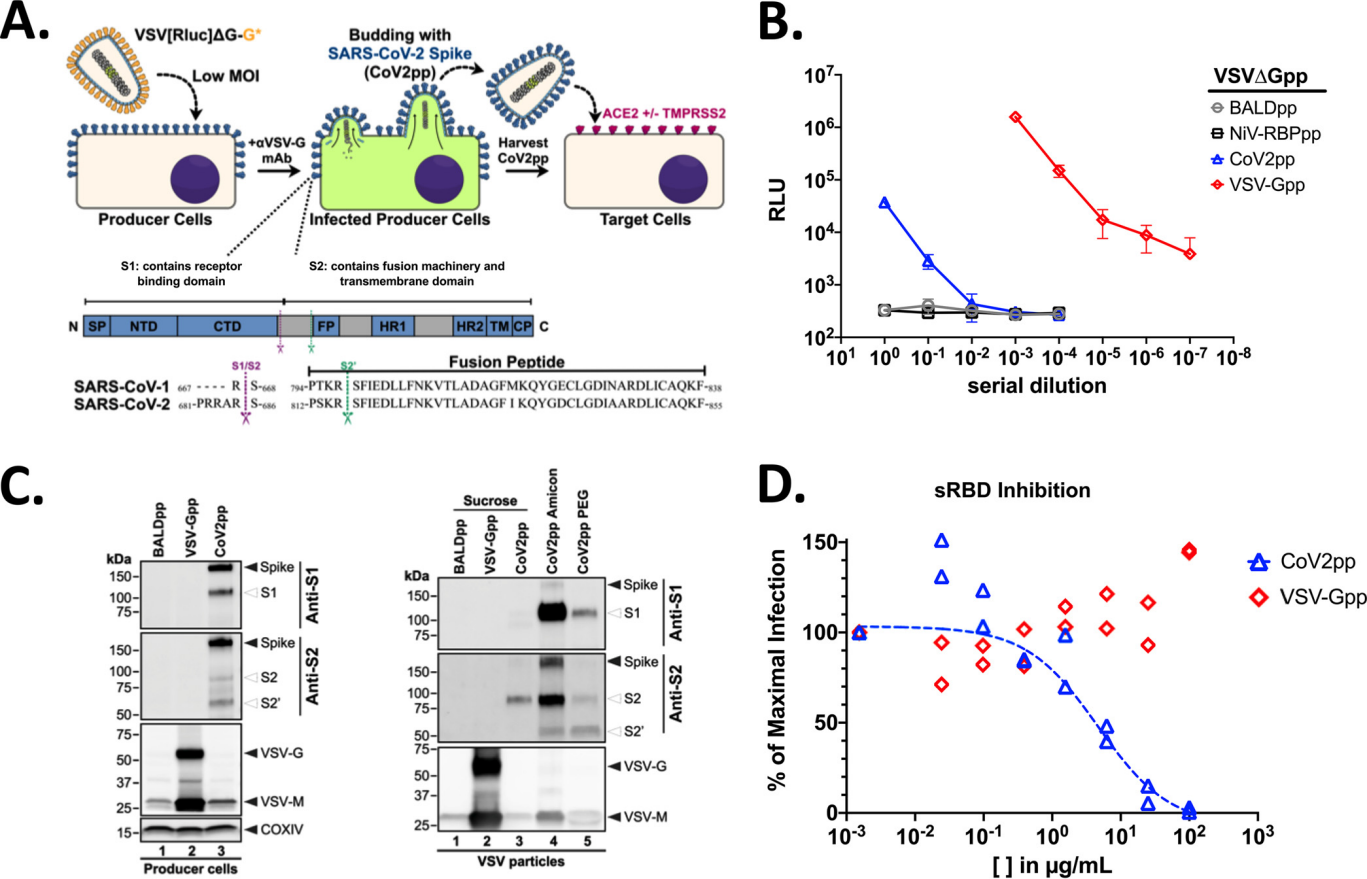
Production of VSVΔG-rLuc bearing the SARS-CoV-2 spike glycoprotein. (A) Overview of VSVΔG-rLuc-pseudotyped particles bearing CoV-2 spike (top), with annotated spike glycoprotein domains and cleavage sites (bottom). As mentioned in the text, we refer to SARS-CoV as SARS-CoV-1 for greater clarity. (B) The titers of VSV-ΔG[Rluc]-pseudotyped particles (VSVpp) bearing the Nipah virus receptor binding protein alone (NiV-RBPpp), SARS-CoV-2-S (CoV2pp), or VSV-G (VSV-Gpp) were determined on Vero-CCL81 cells using a 10-fold serial dilution. Symbols represent the means ± standard errors of the means (SEM) (error bars) from each titration performed in technical triplicates. (C) Expression of the indicated viral glycoproteins on producer cells and their incorporation into VSVpp. Western blots performed as described in Materials and Methods using anti-S1- or anti-S2-specific antibodies. (D) CoV2pp entry is inhibited by the soluble receptor binding domain (sRBD) derived from SARS-CoV-2-S. CoV2pp and VSV-Gpp infection of Vero-CCL81 cells was performed as described for panel B in the presence of the indicated amounts of sRBD. Neutralization curves were generated by fitting data points using a variable slope, a 4-parameter logistics regression curve (robust fitting method). The last point (no sRBD) was fixed to represent 100% maximal infection. The results of each replicate from an experiment performed in duplicate are shown. The calculated IC_50_ for sRBD neutralization of CoV2pp is 4.65 μg/ml.

Our initial objective was to produce SARS-CoV-2 PsV sufficient for ≥10,000 infections/week at an ∼1:100 signal/noise ratio when the assay was performed in a 96-well format. We settled on a VSV-based rather than a lentiviral PsV system, as lentiviruses are intrinsically limited by their replication kinetics and particle production rate (10^4^ to 10^6^/ml for lentiviruses versus 10^7^ to 10^9^/ml for VSV without concentration). Additionally, one advantage of a VSV-based neutralization assay over a lentiviral PsV system is the potential to use them in areas with high HIV prevalence without concerns that the assay may be impacted by patients taking antiretroviral drugs. We optimized the production of our VSVΔG-rLuc pseudotyped viral particles (pp) bearing the SARS-CoV-2 spike glycoprotein as diagramed in Fig. 1A. A detailed production protocol is given in Text S1 in the supplemental material. Notably, this protocol involves infecting producer cells at a low multiplicity of infection (MOI) of stock VSVΔG-G*, incubating producer cells with an anti-VSV-G monoclonal antibody, and generating the pseudotyped particles in Opti-MEM media. The first two measures effectively eliminated the background signal from residual VSV-G, while the last measure allowed for more cleavage of SARS-CoV-2pp in producer cells (Fig. S1). While others have shown that truncating the cytoplasmic tail of SARS-CoV-2-S is typically required for greater functional incorporation into heterologous viral cores (7, 9, 92, 93), we chose to optimize pseudotyping with full-length SARS-CoV-2 spike. Cytoplasmic tail truncations in many other class I viral fusion proteins, including other ACE2-using coronaviruses (HCoV NL63 and SARS-CoV-1), can affect ectodomain conformation and function (94–103). Until such time that we gain a fuller understanding of SARS-CoV-2 entry, we felt that it was necessary to have a surrogate assay that reflects the biology of the full-length virus spike protein.

10.1128/mBio.02492-20.1 TEXT S1 Detailed protocols for the production of VSVΔG bearing the SARS-CoV-2 spike glycoprotein, for use in titering and for use with neutralizations. Download Text S1, DOCX file, 0.6 MB. Copyright © 2021 Oguntuyo et al. 2021 Oguntuyo et al. https://creativecommons.org/licenses/by/4.0/ This content is distributed under the terms of the Creative Commons Attribution 4.0 International license.

10.1128/mBio.02492-20.2 FIG S1 Expression of spike glycoproteins in different growth media. Expression of CoV-2 spike in producer cells shows modestly increased cleavage in the presence of reduced or absent FBS. Western blots were performed as described in Materials and Methods. Download FIG S1, TIFF file, 1.7 MB. Copyright © 2021 Oguntuyo et al. 2021 Oguntuyo et al. https://creativecommons.org/licenses/by/4.0/ This content is distributed under the terms of the Creative Commons Attribution 4.0 International license.

Following the protocol detailed in Text S1, we produced BALDpp, NiV receptor binding protein pp (NiV-RBPpp), CoV2pp, and VSV-Gpp using the VSVΔG-rLuc reporter backbone and determined their titers on Vero-CCL81 cells (Fig. 1B). High-background problems have resulted in low signal/noise ratios when using VSV-based PsV, especially for viral envelope proteins that do not mediate efficient entry. Here, we used two different negative controls, BALDpp and NiV-RBP, to show that we resolved the background issue. BALDpp lacks any surface glycoprotein, while NiV-RBPpp incorporates the NiV-RBP, which binds to broadly expressed ephrin-B2 with subnanomolar affinity (88, 104). However, the NiV fusion (NiV-F) glycoprotein necessary for viral entry is absent. NiV-RBPpp without NiV-F should not fuse and effectively serves as a stricter and complementary negative control. Under the conditions shown, neither BALDpp nor NiV-RBPpp gives any background even at the highest concentration of virus particles used.

These constructs were used to infect Vero-CCL81 cells, and as expected, we observed an average of <500 relative luciferase units (RLUs) of entry with our BALDpp and NiV-RBPpp negative controls. These levels of entry were comparable to the “cells only” signal, providing confidence in any infection signals 10-fold over background. Undiluted CoV2pp entry resulted in luciferase values of over 50,000 RLUs, greater than 100-fold over background BALDpp signals (Fig. 1B). VSV-Gpp gave infectivity several logs higher, as expected. Western blots of the producer cells demonstrated effective expression of cleaved SARS-CoV-2 spike glycoproteins (Fig. 1C, left panel). Cleaved CoV-2 spike products (S1, S2, and S2′) all appear to be incorporated into the VSVΔG pseudotyped particles (Fig. 1C, right panel). To ensure that entry of CoV2pp is SARS-CoV-2 spike mediated, we show that the homologous soluble receptor binding domain (sRBD) of spike competitively inhibits our CoV2pp (Fig. 1D).

### CoV2pp entry is enhanced by trypsin treatment and spinoculation.

**FIG 2:**
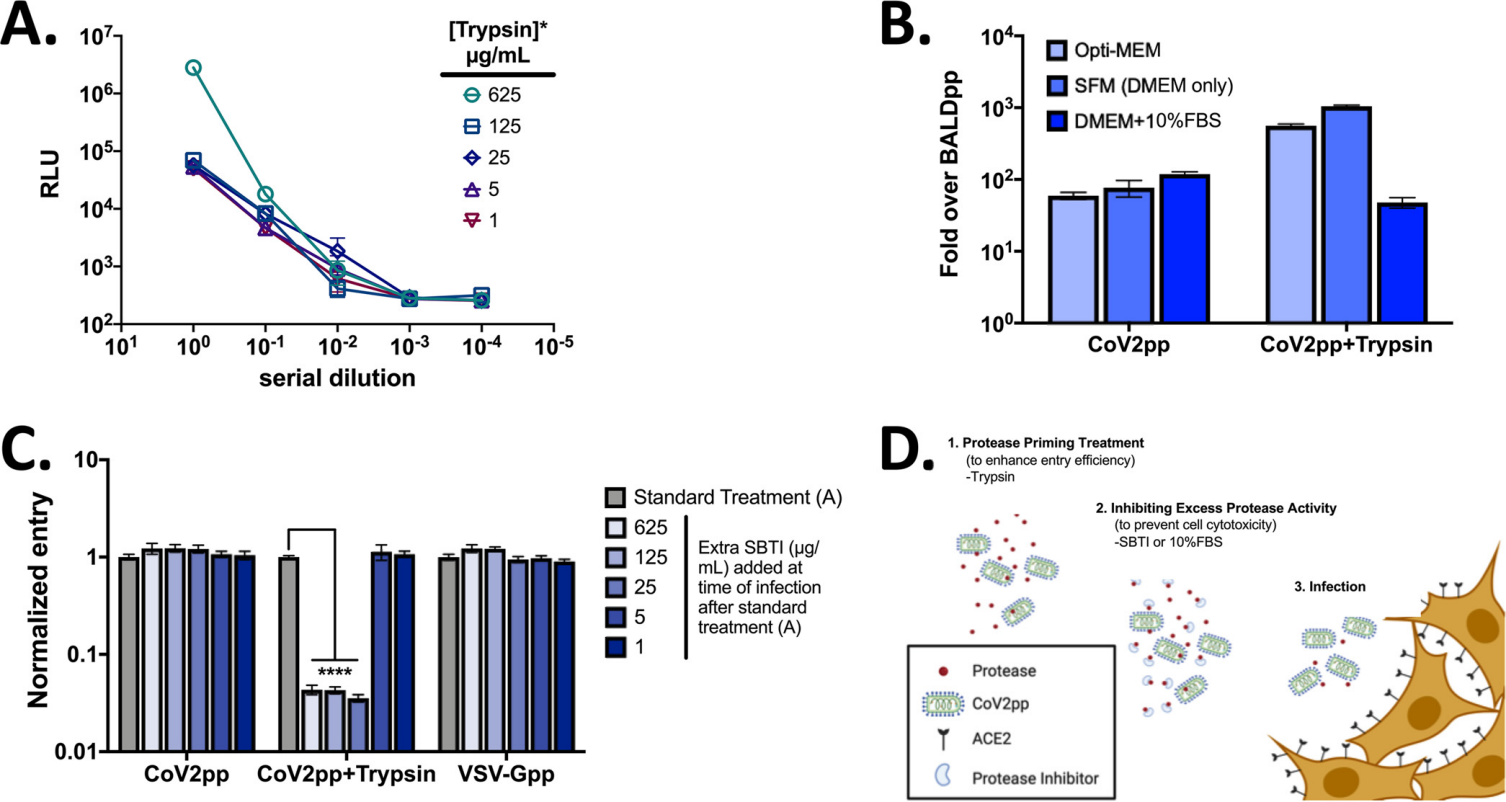
CoV2pp entry is enhanced by trypsin treatment. (A) Optimizing trypsin treatment conditions. Supernatants containing CoV2pp were trypsin treated at the indicated concentrations for 15 min at room temperature prior to the addition of 625 μg/ml of SBTI. The titers of these particles were then determined on Vero-CCL81 cells in technical triplicates. Data are shown as means ± SEM. (B) Dilution in serum-free media (SFM, DMEM only) provides the highest signal/noise ratio for trypsin-treated CoV2pp entry. Particles were diluted 1:10 in Opti-MEM, SFM, or DMEM plus 10% FBS prior to infection of Vero-CCL81 cells and spinoculation as described in the legend of Fig. 1D. Cells infected without spinoculation show approximately 3×-lower signal/noise ratios (see Fig. S2 in the supplemental material). (C) Addition of soybean trypsin inhibitor at the time of infection reduces trypsin-treated particle entry. This assay was performed in technical triplicates for two independent experiments. Shown are the combined results, with error bars indicating SEM and **** indicating a *P* value of <0.0001. (D) Schematic showing an overall view of how protease priming and SBTI treatment works to enhance CoV2pp entry.

Next, we sought to enhance the relative signal of our CoV2pp infections, which effectively increases the number of infections that we can provide or perform per batch of CoV2pp. Trypsin treatment is reported to enhance SARS-CoV-1 and SARS-CoV-2 entry (39, 45). Thus, we treated CoV2pp stocks with the indicated range of trypsin concentrations for 15 min at room temperature (Fig. 2A). In order to mitigate the effects of trypsin-dependent cytotoxicity, we added 625 μg/ml of soybean trypsin inhibitor (SBTI) to all samples before titrating the trypsin-treated CoV2pp onto Vero-CCL81 cells. CoV2pp treated with the highest concentration of trypsin (625 μg/ml) resulted in an ∼100-fold enhancement of entry (Fig. 2A), but this trypsin-dependent enhancement was apparent only when we compared entries of undiluted trypsin-treated CoV2pp. We observed a >50-fold reduction in entry (RLUs) after a 10-fold serial dilution, which nullified any entry enhancement effects of trypsin. Indeed, the role of trypsin in enhancing SARS-CoV-2 entry has not been fully determined. Trypsin may act to prime CoV2pp to facilitate better entry upon spike-receptor interactions and/or assist to proteolytically activate spike protein at or after receptor binding (50). We hypothesized that the remaining uninhibited trypsin-dependent effect, which must be present at the highest trypsin concentration, was inadvertently neutralized by diluting the trypsin-treated CoV2pp in Dulbecco’s modified Eagle medium (DMEM) plus 10% fetal bovine serum (FBS), which is the standard infection medium for titrating CoV2pp. To test this hypothesis, we diluted CoV2pp and trypsin-treated CoV2pp 1:10 under three different medium conditions before infecting Vero-CCL81 cells. For trypsin-treated CoV2pp, dilution in DMEM alone (serum-free medium [SFM]) produced the highest signal/noise ratio, almost 1,000-fold over that of BALDpp (Fig. 2B). As a result, we chose CoV2pp treated with 625 μg/ml of TPCK (tosylsulfonyl phenylalanyl chloromethyl ketone)-treated trypsin and then 625 μg/ml of SBTI diluted in SFM as our standard treatment condition. Furthermore, spinoculation at 1,250 rpm for 1 h enhanced entry 3- to 5-fold (compare signal/noise ratios in Fig. 2B to those in Fig. S2).

10.1128/mBio.02492-20.3 FIG S2 Dilution of CoV2pp in the absence of serum-free media produces the highest signal/noise ratios for trypsin-treated CoV2pp. Dilution was performed as described in Fig. 2B but was done in the absence of spinoculation. Presented are the results from an experiment in technical triplicates; error bars show the SEM. Download FIG S2, TIFF file, 0.5 MB. Copyright © 2021 Oguntuyo et al. 2021 Oguntuyo et al. https://creativecommons.org/licenses/by/4.0/ This content is distributed under the terms of the Creative Commons Attribution 4.0 International license.

Our above hypothesis suggests that the uninhibited trypsin-dependent enhancing effect acts at the point of infection when CoV2pp interact with the host cell receptor. To investigate further, we spiked additional SBTI onto cells at the time of infection using particles produced under the standard treatment condition, as above. We found that additional SBTI (≥25μg/ml) added directly to cells at the point of infection was able to inhibit trypsin-dependent entry enhancement (Fig. 2C). The data suggest that some trypsin was not inhibited by the first 625 μg/ml of SBTI and that enough remained to enhance entry at the point of infection (Fig. 2D).

### Entry of CoV2pp is independently enhanced by stable expression of ACE2 and TMPRSS2 in cells already permissive for SARS-CoV-2 entry and replication.

**FIG 3:**
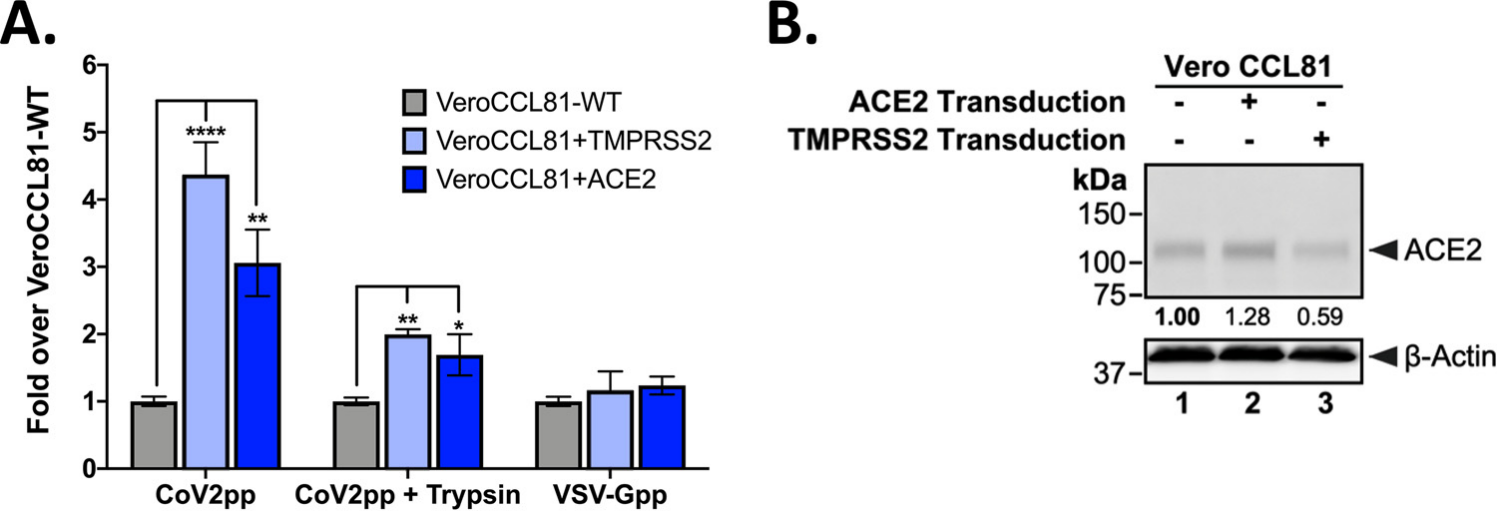
Trypsin-treated CoV2pp depend on ACE2 and TMPRSS2 for entry. (A) Parental and TMPRSS2- or ACE2-transduced Vero-CCL81 cells were infected with the indicated pseudotyped viruses. All particles were diluted in serum-free media in order to be within the linear range for the assay. Normalized infectivity data are presented as fold values over those for Vero-CCL81 WT cells for the various VSVpp shown. VSV-Gpp served as an internal control for the intrinsic permissiveness of various cell lines to VSV-mediated gene expression. Data are presented as means ± SEM from two independent experiments done in technical triplicates. *, *P* < 0.05; **, *P* < 0.01; ****, *P* < 0.0001. (B) Western blot of wild-type and transduced Vero CCL81 cells. The numbers below each column show that the relative ACE2 abundance was measured by densitometry and normalized as described in Materials and Methods.

To further characterize the determinants of CoV2pp entry, we generated Vero-CCL81 cell lines stably expressing human ACE2 or human TMPRSS2. Vero-CCL81 cells are already highly permissive for SARS-CoV-2 entry and replication. We infected the indicated cells with CoV2pp or trypsin-treated CoV2pp diluted in serum-free medium (standard treatment) and observed enhanced entry in both stable cell lines (Fig. 3A). However, the entry enhancement of trypsin-treated CoV2pp in Vero-CCL81 TMPRSS2-overexpressing cells was subdued relative to that in untreated CoV2pp. This suggests that the presence of exogenous trypsin during CoV2pp entry can substitute, in part, for the role played by cell surface TMPRSS2, an endogenous protease known to facilitate entry into physiologically relevant cell types *in vivo* (40). Figure 3B shows that the relationship between ACE2 and TMPRSS2 expression—with regard to their effect on enhancing SARS-CoV-2 spike-mediated entry—is not straightforward. As ACE2 itself is a substrate for TMPRSS2, the right stoichiometry of receptor/protease expression appears to be the main driver of entry efficiency rather than the absolute expression of one or the other. This issue will be further examined in the last section.

### Standardizing the parameters that impact our CoV2pp-based virus neutralization assay.

Having established that exogenous trypsin can serve as a physiologically relevant substitute for endogenous proteases known to enhance entry of CoV2pp, such as TMPRSS2, we sought to characterize the parameters that might affect the performance our CoV2pp VNA. Conditions tested included heat inactivation of sera and the infection medium used to dilute human serum samples. We used representative spike ELISA-positive or -negative sera to serve as positive and negative controls, respectively. When sera were first diluted in SFM, we observed that negative sera can have alarming amounts of neutralizing activity that appear specific for CoV2pp, as the same sera did not neutralize VSV-Gpp entry (compare Fig. S3A with Fig. S3B, right panel). This CoV2pp serum-neutralizing factor is somewhat reduced but not completely diminished by heat inactivation for 1 h at 56°C. Notably, the effect of this neutralizing factor from negative sera was preempted by diluting the trypsin-treated CoV2pp in DMEM containing 10% FBS (Fig. S3B). Importantly, recombinant sRBD neutralization was not affected by the dilution of CoV2pp in SFM or DMEM plus 10% FBS (Fig. S3C). The nature of the factor that appears to inhibit spike-mediated entry is the subject of a concurrent manuscript in submission (see Discussion) (105). Regardless, for standardizing our CoV2pp-based VNA, all subsequent patient sera were heat inactivated for at least 30 min prior to use and serially diluted in DMEM plus 10% FBS, which also served as our infection medium. Despite our data from Fig. 2 implicating a trypsin inhibitor-like activity in FBS, the marked inhibition of CoV2pp entry by seronegative human sera is a greater limiting factor that prevents the robust determination of true SARS-CoV-2 neutralizing antibody (nAb) titers. To achieve the same signal/noise ratio while performing our VNA in the presence of 10% FBS, we increased the concentration of CoV2pp used per infection.

### Performance characteristics of our standardized CoV2pp virus neutralization assay.

**FIG 4:**
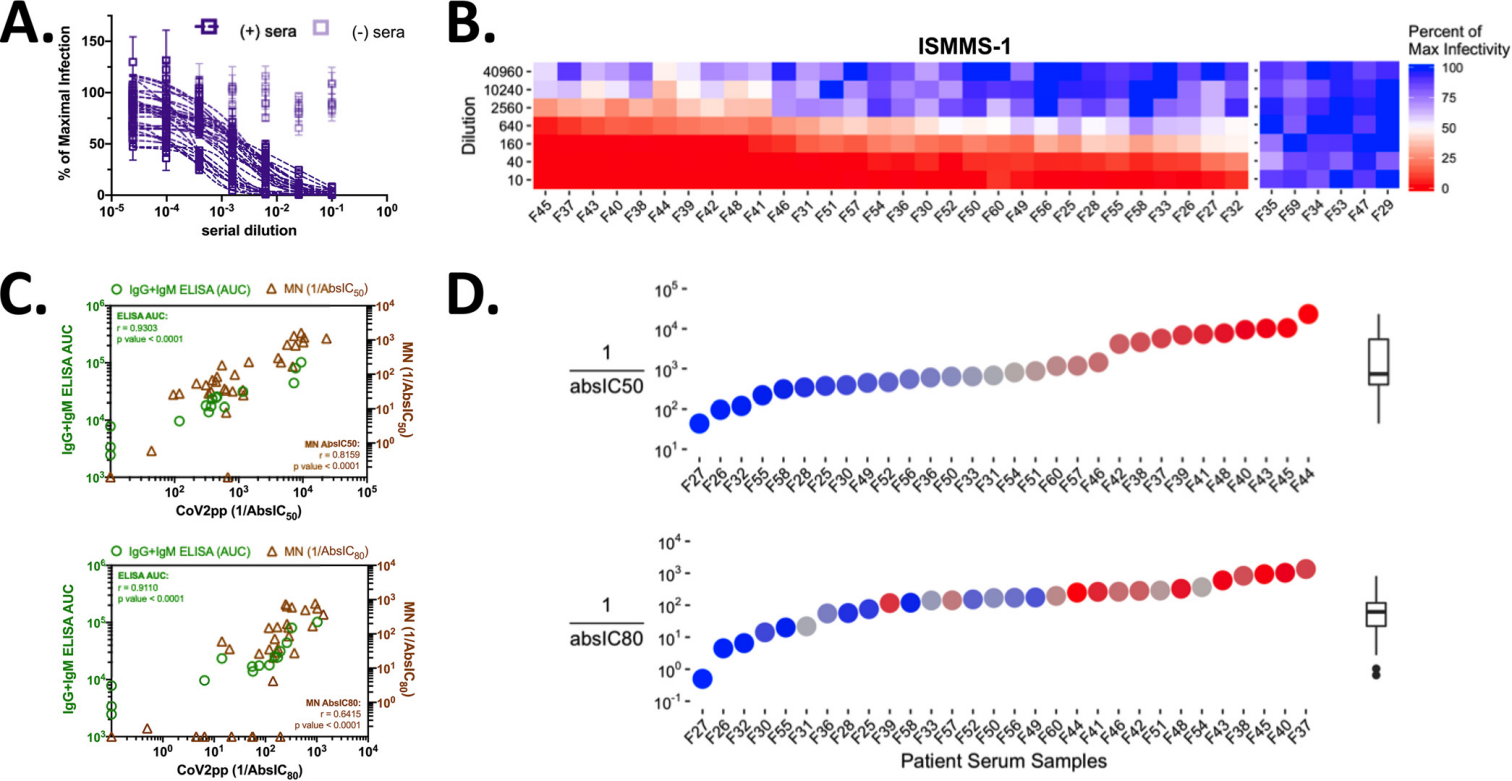
CoV2pp viral neutralization assay and absIC_50_/absIC_80_ values versus those after spike binding of patient sera. (A) Thirty-six patient sera screened for CoV2pp neutralization. CoV2pp were used to infect Vero-CCL81 cells in the presence of a 4-fold serial dilution of patient sera, as described in Materials and Methods. Samples in light purple do not neutralize CoV2pp. Neutralization curves were fit using a variable-slope, 4-parameter logistics regression curve with a robust fitting method. (B) The same 36 samples are shown as a neutralization heat map, which was generated in R as described in Materials and Methods. Here, red represents complete neutralization, and blue represents no neutralization. Samples are sorted by the average from the first four dilutions, with the most neutralizing samples on the left. (C) Correlation of CoV2pp neutralization titers to spike binding (ELISA AUC, green circles) and live-virus microneutralization (MN, brown triangles) activity. The absolute IC_50_ (absIC_50_, top) and IC_80_ (absIC_80_, bottom) for CoV2pp neutralizations and live-virus MNs were calculated in R using a 4-parameter logistic regression model as described in Materials and Methods. Presented are the added IgG and IgM ELISA AUCs. AUC determinations and live-virus neutralizations were performed as described in Materials and Methods. Presented are the *r* and *P* values from a simple linear regression. (D) Positive serum samples and their CoV2pp reciprocal absIC_50_ (top) and absIC_80_ (bottom) values. The IC_50_ graph is colored and ordered to display samples with low, average, or high IC_50_s as blue, gray, or red circles, respectively. The IC_80_ graph below retains the coloring from the IC_50_ graph, but the samples are now ordered from left to right to show samples with the lowest to highest IC_80_ values. Tukey box and whisker plots show medians with interquartile ranges (IQR) and whiskers extending to 1.5× the IQR. All points outside that range are depicted.

An initial set of sera for validation of CoV2pp VNA was generously provided by Florian Krammer. These sera were screened according to a previously described two-stage ELISA protocol in which 1:50 dilutions of patient sera were first screened for reactivity against the sRBD. Subsequently, the presumptive RBD-positive patient sera were used to assess reactivity to the trimer-stabilized ectodomain of spike at five different dilutions (1:80, 1:160, 1:320, 1:960, and 1:2,880) (73, 106). These samples were used for neutralization studies with CoV2pp (Fig. 4A and B). From the 36 patient sera tested, 6 were found to be negative for SARS-CoV-2 spike binding in the ELISA described above. All of those 6 serum samples also showed no neutralization of CoV2pp. The remaining 30 spike-positive sera had 50% neutralizing titers that span 2 orders of magnitude (Fig. 4B, 160 to 10,240). For a more quantitative assessment of the correlation of our assay to the other standards in the field, we compared the nAb titers as determined by our CoV2pp VNA to both spike binding activity and live-virus microneutralization (MN) titers for identical serum samples. We determined the total IgG and IgM spike binding activity (ELISA area under the concentration-time curve [AUC], as described in Materials and Methods) of a representative subset of 15 serum samples and compared them with their reciprocal absIC_50_ and absIC_80_ values, calculated from the CoV2pp neutralization curves (Fig. 4A), as described in Materials and Methods. Spike binding antibodies (IgG plus IgM ELISA AUC) demonstrated a significant, positive correlation with nAb titers, as determined by our CoV2pp VNA (Fig. 4C, green circles). Moreover, these nAb titers against CoV2pp also correlated well with live-virus MN titers (MN absIC_50_, MN absIC_80_) (Fig. 4C, brown triangles). Full neutralization curves for the MN titers are shown in Fig. S4. absIC_80_ appeared to be a more stringent measure of nAb activity, as some sera that have respectable MN absIC_50_ titers never achieve an absIC_80_ (Fig. 4C, bottom graph, brown triangles on the *x* axis, and Fig. S4). This is due in part to the difference in the dynamic ranges between a luciferase-based assay (≥3 logs RLUs) and an MN assay (∼1.5-log optical density [OD] values corresponding to the amount of viral protein detected). The larger dynamic range of the CoV2pp VNA was thus more able to resolve the serum samples that can reach their respective absIC_80_ values. Notably, we find that serum samples with potent absIC_50_ titers do not always display potent absIC_80_ values (Fig. 4D).

10.1128/mBio.02492-20.4 FIG S3 Serum neutralization in the absence of 10% FBS and optimization of neutralizations. (A) Negative serum potently inhibits trypsin-treated CoV2pp. CoV2pp were diluted in serum-free medium (SFM), and then pooled negative sera and a positive serum were used to neutralize entry. An aliquot was heat inactivated (HI) for 1 h in a 56°C water bath prior to use. Neutralizations were performed as described in Materials and Methods. Data are presented on linear (left) and log (right) scales. Each replicate from one experiment in technical duplicates is shown, and neutralization curves were generated as done for Fig. 1D. (B) Serum neutralizations were performed with untreated CoV2pp (left) or CoV2pp treated with trypsin (middle). Both particles were diluted in DMEM plus 10% FBS, and neutralization curves are presented as described above. VSV-G was not neutralized by the negative or positive sera (right). (C) sRBD neutralizes CoV2pp equivalently across all conditions tested. Data presented in Fig. 1D (i.e., the untreated CoV2pp) are duplicated here. Neutralization curves are presented as described above. Download FIG S3, TIFF file, 2.5 MB. Copyright © 2021 Oguntuyo et al. 2021 Oguntuyo et al. https://creativecommons.org/licenses/by/4.0/ This content is distributed under the terms of the Creative Commons Attribution 4.0 International license.

**FIG 5:**
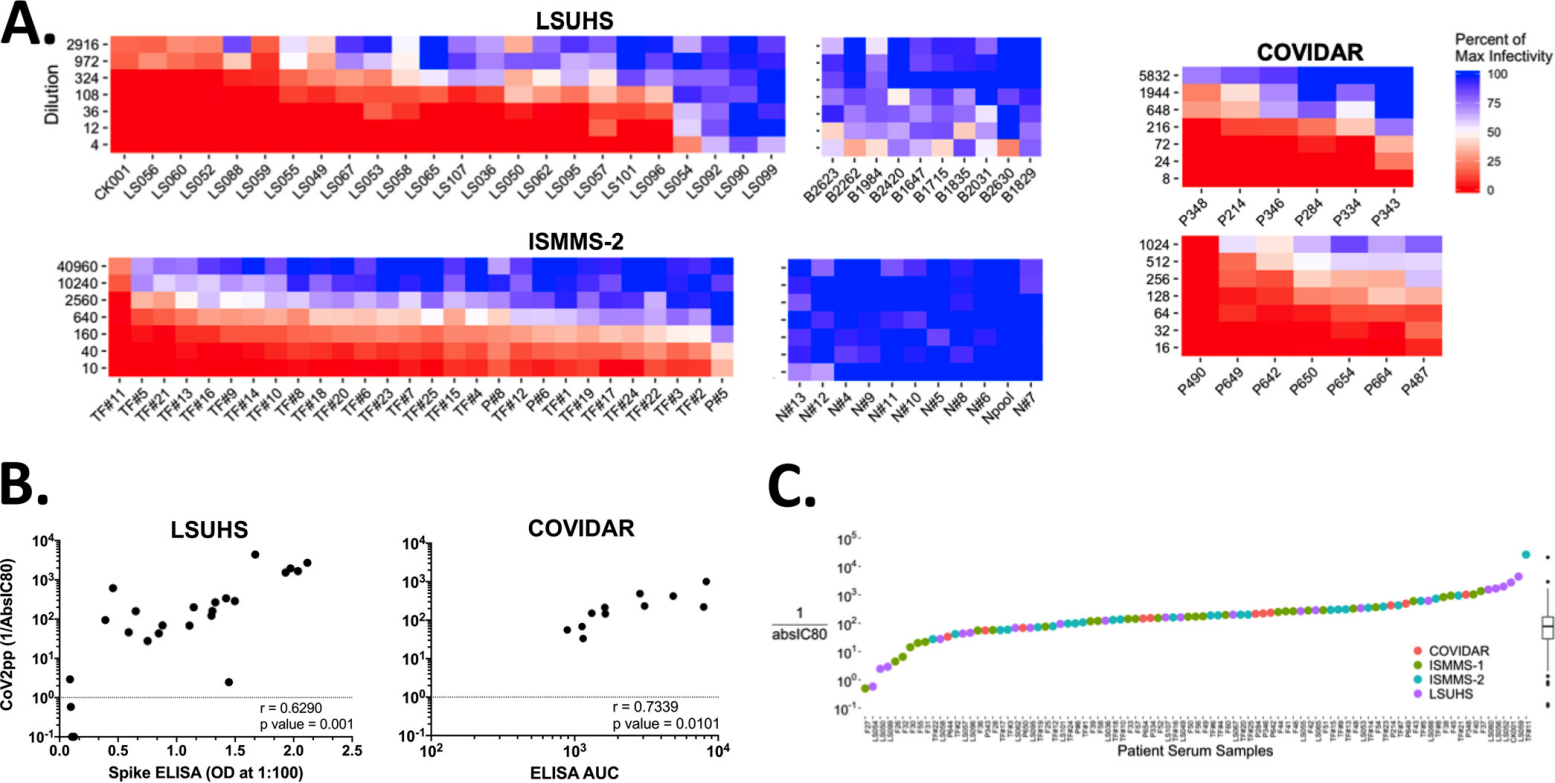
CoV2pp viral neutralization assay validated against patient sera by external groups. (A) Patient serum neutralization of CoV2pp for 88 samples run by three different independent groups. This is visualized as in Fig. 4B, where red represents complete neutralization and blue represents no neutralization. (B) Correlations of CoV2pp reciprocal absIC_80_s to those of spike ELISAs. The absIC_80_ was calculated as described in the legend of Fig. 4C. For LSUHS ELISAs, the spike ectodomain was used and sera were diluted to a 1:100 dilution. For the COVIDAR ELISAs, a mixture of the sRBD and spike was utilized as previously described (116), and the AUC was calculated as described in Materials and Methods. (C) Summary absIC_80_s of 89 positive serum CoV2pp neutralizations. Samples from all 4 groups are depicted on the *x* axis. The absIC_80_ was calculated as described in the legend of Fig. 4C, and Tukey box and whisker plots are shown as described in the legend of Fig. 4D.

10.1128/mBio.02492-20.5 FIG S4 All full-neutralization curves. (A) Live SARS-CoV-2 microneutralization curves for ISMMS-1. Live-virus microneutralization curves were determined as described in Materials and Methods. Neutralizations were performed in technical duplicates, and shown are standard deviations (SD). Data are presented as in Fig. 4A and fit to a variable-slope, 4-parameter logistics curve. (B) Neutralization curves from the LSUHS, ISMMS-2, and COVIDAR labs. The neutralization curves presented here were generated from the same data used to create the neutralization heat maps shown in Fig. 5A. The curves were fit using a variable-slope, 4-parameter logistics model (robust regressions fitting). The ISMMS-2 group (top left panel) and COVIDAR group (bottom panels) performed neutralizations in technical triplicates. The LSUHS group (top right panel) performed their neutralizations in technical quadruplicates. Download FIG S4, TIFF file, 3.5 MB. Copyright © 2021 Oguntuyo et al. 2021 Oguntuyo et al. https://creativecommons.org/licenses/by/4.0/ This content is distributed under the terms of the Creative Commons Attribution 4.0 International license.

### Independent validation of our CoV2pp VNA with geographically distinct and ethnically diverse COVID-19 patient cohorts.

To assess the robustness of our standardized CoV2pp VNA, we produced and distributed the CoV2pp to many labs who have requested our assay for use in various screens for nAbs. Here, we analyze and present the raw virus neutralization data provided to us by three independent groups at the Icahn School of Medicine at Mount Sinai (ISMMS), at the Louisiana State University Health Sciences Center Shreveport (LSUHS), and in Argentina (COVIDAR). In serum or plasma neutralization studies performed in these independent labs, these groups also observed similar absIC_50_, absIC_80_, and absIC_90_ distributions. The LSUHS and ISMMS group 2 (ISMMS-2) cohorts represent data from 25 and 28 seropositive as well as 10 and 11 seronegative samples, respectively, while the COVIDAR consortium assessed neutralization from an initial set of 13 seropositive patient samples. For clarity, an analysis of their neutralization curves is presented as heatmaps in Fig. 5A, similar to what is shown in Fig. 4B. Full neutralization curves for each cohort are shown in Fig. S4.

The seronegative control samples from all groups revealed no CoV2pp neutralization. Rare, but notable, seropositive samples from LSUHS also showed no neutralization (Fig. 5A, LSUHS). ISMMS-2 performed its analysis on confirmed convalescent-phase plasma donors (32). While all donors had detectable nAb titers, their titers were highly varied and ranged across 2 to 3 logs. absIC_80_s were calculated for all samples shown, and we observed a moderate, but significant, positive correlation between various spike ELISA metrics and absIC_80_s (Fig. 5B).

Aggregated reciprocal absIC_80_s from all three external labs as well as our own are shown in Fig. 5C. In total, we analyzed absIC_80_s from 89 individuals across four groups. The descriptive statistics from this aggregated data set reveals reciprocal absIC_80_s in a 25th percentile of 68.5, a median of 170.8, and a 75th percentile of 343.4. Descriptive statistics for reciprocal absIC_50_ and absIC_90_ values were also calculated and are reported in Table S1. Using the absIC_80_ descriptive statistics above and the ELISA endpoint titers from our initial 36 serum samples, we observe that 0% of the samples displaying an ELISA endpoint titer of 320 have an absIC_50_ greater than the median IC_50_. Perhaps not surprisingly, over 90% of samples with ELISA endpoints of 2,880 have IC_50_s at or beyond the 75th percentile (Table 1; represented graphically in Fig. S5). Although the absIC_80_ also generally follows this trend, we once again note differences in ranked order of absIC_50_ and absIC_80_ values calculated for all serum samples. This difference is more pronounced when comparing the absIC_50_ and absIC_90_ graphs, further highlighting the need for a neutralization assay with a broad dynamic range (Fig. S7, left panel). Additionally, the samples from each of the 4 groups show no statistical difference when absIC_50_, absIC_80_, or absIC_90_ calculations are compared (Fig. S7, right panel). All together, these data support the robustness of our CoV2pp VNA and suggest that the absIC_80_ is a more stringent and meaningful measure of nAb titers.

**TABLE 1:** Comparison of ELISA endpoint titers to CoV2pp neutralization^*a*^

ELISA endpoint	IC_50_ summary (fraction of samples [%])			IC_80_ summary (fraction of samples [%])		
	≥25th percentile	≥50th percentile	≥75th percentile	25th percentile	≥50th percentile	≥75th percentile
320	7/11 (63.6)	**0/11 (0)**	0/11 (0)	4/11 (36.4)	1/11 (9.1)	0/11 (0)
960	6/8 (75)	4/8 (50)	0/8 (0)	7/8 (87.5)	4/8 (50)	1/8 (12.5)
2,880	11/11 (100)	11/11 (100)	**10/11 (90.9)**	11/11 (100)	10/11 (90.9)	**5/11 (45.5)**

a Presented are the clinical lab ELISA endpoint titers from samples discussed in Fig. 4A. Descriptive statistics were generated in PRISM using data presented in Fig. 5C. Values highlighted in bold are of interest and are discussed further in Results.

10.1128/mBio.02492-20.6 FIG S5 Relationship of ELISA endpoint titers and CoV2pp reciprocal absIC_50_, absIC_80_, and absIC_90_ values. Presented are the clinical lab ELISA endpoint titers and CoV2pp neutralization absIC values. There are 11 samples with an ELISA endpoint of 320, 8 samples with an ELISA endpoint of 960, and 11 samples with an ELISA endpoint of 2,880. absIC_50_, absIC_80_, and absIC_90_ values were calculated as described in Materials and Methods. Error bars (blue) show median and interquartile ranges; red and black dotted lines represent the median and 75th percentile for each absIC value, as calculated in Table S1 in the supplemental material. The gray-shaded region indicates samples that fall above the 75th percentile. One sample with an ELISA endpoint of 320 had an absIC_90_ below 10^−1^ and thus is not present on the absIC_90_ graph. Download FIG S5, TIFF file, 1.0 MB. Copyright © 2021 Oguntuyo et al. 2021 Oguntuyo et al. https://creativecommons.org/licenses/by/4.0/ This content is distributed under the terms of the Creative Commons Attribution 4.0 International license.

10.1128/mBio.02492-20.8 FIG S7 CoV2pp absolute IC_50_ (top), IC_80_ (middle), and IC_90_ (bottom) plots from all four groups. Left panels present ordered IC values as previously described for Fig. 4D and in Materials and Methods. Right panels display a comparison of CoV2pp absolute IC_50_, IC_80_, and IC_90_ values across all 4 groups, which reveals no statistically significant difference. Groups were compared using an ordinary one-way analysis of variance (ANOVA) with Dunnett’s correction for multiple comparisons (n.s., *P* value ≥ 0.05). Download FIG S7, TIFF file, 4.6 MB. Copyright © 2021 Oguntuyo et al. 2021 Oguntuyo et al. https://creativecommons.org/licenses/by/4.0/ This content is distributed under the terms of the Creative Commons Attribution 4.0 International license.

10.1128/mBio.02492-20.10 TABLE S1 Descriptive statistics for CoV2pp neutralizations across 4 groups. Presented are the descriptive statistics from the CoV2pp neutralizations shown in Fig. 5C. Absolute IC_50_, IC_80_, and IC_90_ values were calculated as described in Materials and Methods. Median and other percentiles presented here were calculated in PRISM. Download Table S1, TIFF file, 0.7 MB. Copyright © 2021 Oguntuyo et al. 2021 Oguntuyo et al. https://creativecommons.org/licenses/by/4.0/ This content is distributed under the terms of the Creative Commons Attribution 4.0 International license.

### Ultrapermissive 293T-ACE2 and 293T-ACE+TMPRSS2 clones allow for use of CoV2pp in VNA at scale.

Although our standardized VNA appears robust, the requirement for exogenous trypsin and spinoculation to achieve the optimal signal/noise ratio limits the scalability of our VNA. Therefore, we used our untreated CoV2pp to screen for ultrapermissive cell lines that would allow for our CoV2pp VNA to be performed with dilutions of virus supernatant without any trypsin treatment, virus purification, or spinoculation.

**FIG 6:**
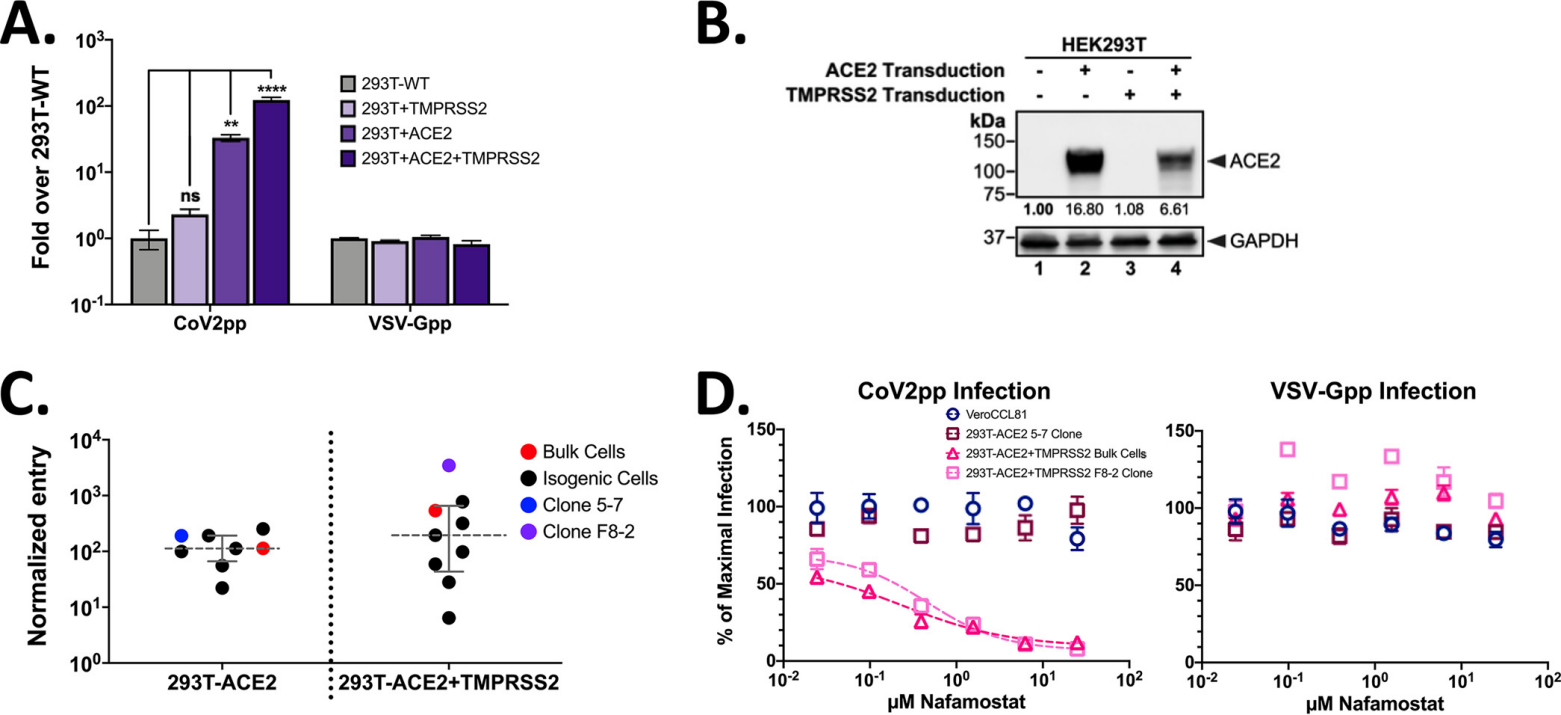
293T cells stably transduced with ACE2 and TMPRSS2 (293T-ACE2+TMPRSS2) are ultrapermissive for SARS-CoV-2pp infection. (A) Infection of 293T cell lines transduced to stably express TMPRSS2, ACE2, or both. Cell lines were generated as described in Materials and Methods. A single dilution of particles was used to infect cells prior to spinoculation as described in Materials and Methods. Infections were done in technical triplicates. Presented are the aggregated results from two independent replicates, and error bars show SEM. For statistics, ns indicates not significant, ** indicates a *P* value of <0.01, and **** indicates a *P* value of <0.0001. (B) Western blot of ACE2 expression in 293T cell lines. Blotting was performed as described in Materials and Methods, and the values below the columns represent the relative abundances of ACE2. (C) Normalized CoV2pp entry into single-cell clones. Entry was normalized to that of the wild-type parental cell line and further normalized to VSV-G entry. Presented are the averages from one experiment in technical triplicates. Error bars show the medians and interquartile ranges. Raw entry data for each cell clone are shown in Fig. S8A. (D) Entry inhibition of CoV2pp by nafamostat mesylate, a serine protease inhibitor. Nafamostat was mixed with CoV2pp (left panel) or VSV-Gpp (right panel) prior to addition to cells. Shown are the results from one experiment in technical triplicates. Data are presented as described in the legend of Fig. 4A, and error bars show SEM.

We generated three different 293T cell lines stably expressing ACE2 and/or TMPRSS2 via lentiviral transduction. We then infected these cells with CoV2pp. Increased expression of TMPRSS2 alone (293T-TMPRSS2) did not significantly improve entry (Fig. 6A), likely due to the low to undetectable ACE2 expression levels (Fig. 6B, lanes 1 and 3). However, expression of ACE2 significantly increased the entry of CoV2pp, which was further increased in 293T-ACE2 plus TMPRSS2 (293T-ACE2+TMPRSS2) cells, suggesting the synergistic activity of TMPRSS2 and ACE2 (Fig. 6A). Western blot analysis confirmed the increased expression of ACE2 in the singly and doubly transduced 293T cells (Fig. 6B). Additionally, increased expression of both ACE2 and TMPRSS2 was confirmed by quantitative PCR (qPCR) (Fig. S8B). Interestingly, ACE2 expression appeared to be decreased by >50% in 293T-ACE2+TMPRSS2 cells relative to that in 293T-ACE2 cells. These observations highlight the complex roles that receptor binding and protease activation play in SARS-CoV-2 entry, especially since ACE2 is a known substrate for TMPRSS2 (107) and TMPRSS2 is also known to undergo autocatalytic cleavage (108).

10.1128/mBio.02492-20.9 FIG S8 Screening and validation of single-cell clones. (A) Raw RLU values from infection of the indicated cells by BALDpp, CoV2pp, or VSV-Gpp. Parental cell lines, bulk-transduced cell lines, and isogenic cell lines are indicated. Highlighted in blue and purple are the ultrapermissive clones stably expressing ACE2 or ACE2 and TMPRSS2, respectively. Presented are the results from an experiment in technical triplicates, and error bars show the SEM. (B) Expression of ACE2 and TMPRSS2 in select cell lines. RNA extraction and qPCR were performed as described in Materials and Methods prior to calculation of the 2^–ΔΔ^*^CT^*, which was then normalized to the 293T parental cells. Of interest are the clones highlighted in blue and purple, which were transduced to stably express ACE2 or ACE2 and TMPRSS2. (C) Titers of CoV2pp were determined on Vero-CCL81 cells (left), 293T-ACE2 clone 5-7 (middle), and 293T-ACE2-TMPRSS2 clone F8-2 (right). Titrations were performed with untreated CoV2pp and without spinoculation. Presented are the results from technical triplicates, and bars show the SEM. Download FIG S8, TIFF file, 3..3 MB. Copyright © 2021 Oguntuyo et al. 2021 Oguntuyo et al. https://creativecommons.org/licenses/by/4.0/ This content is distributed under the terms of the Creative Commons Attribution 4.0 International license.

Given how TMPRSS2 can enhance ACE2-dependent virus entry in a nonlinear fashion, we used BALDpp, CoV2pp, and VSV-Gpp to screen 19 single-cell clones derived from 293T-ACE2, 293T-ACE2+TMPRSS2, or Vero-ACE2 bulk-transduced cells. The last (Fig. 3) served as an additional control in a naturally permissive cell line for SARS-CoV-2 entry and replication. All three bulk-transduced cell lines resulted in significant increases in entry levels of CoV2pp relative to those of the parental 293T and Vero CCL81 cells (Fig. S8 and Fig. 6C). However, only a subset of the single-cell clones performed better than bulk-transduced cells. This is especially notable in single-cell clones derived from 293T-ACE2+TMPRSS2 parental cells, of which only two of eight single-cell clones showed greater entry than the bulk-transduced cells (Fig. 6C). One particular clone, F8-2 (Fig. 6C), showed a nearly 10-fold increase in CoV2pp entry relative to that of the bulk-transduced cells. Using F8-2 to titer untreated CoV2pp without spinoculation, we observed a dramatic increase in signal/noise ratios relative to that of wild-type Vero-CCL81 (Vero-CCL81 WT) cells and even the most permissive 293T-ACE2 clone, 5-7 (Fig. S8C), such that RLU signals were consistently 100- to 200-fold over those of BALDpp even at a 1:50 dilution. TMPRSS2 was determined to be the main driver of this entry enhancement in the F8-2 cells, as treatment with nafamostat, a serine protease inhibitor, potently inhibited entry. However, this entry inhibition plateaued at 90% of maximal infection, and the remaining 10% is nearly equivalent to the raw RLU values seen with bulk 293Ts stably expressing ACE2 alone (Fig. 6D and Fig. S8B), suggesting a TMPRSS2-independent mechanism of entry. Entry into 293T-ACE2 cells was not inhibited by nafamostat, once again highlighting that CoV2pp can enter by both the early and late entry pathways that have differential protease requirements.

### Diverse cell lines maintain similar kinetics in CoV2pp viral neutralization assays.

**FIG 7:**
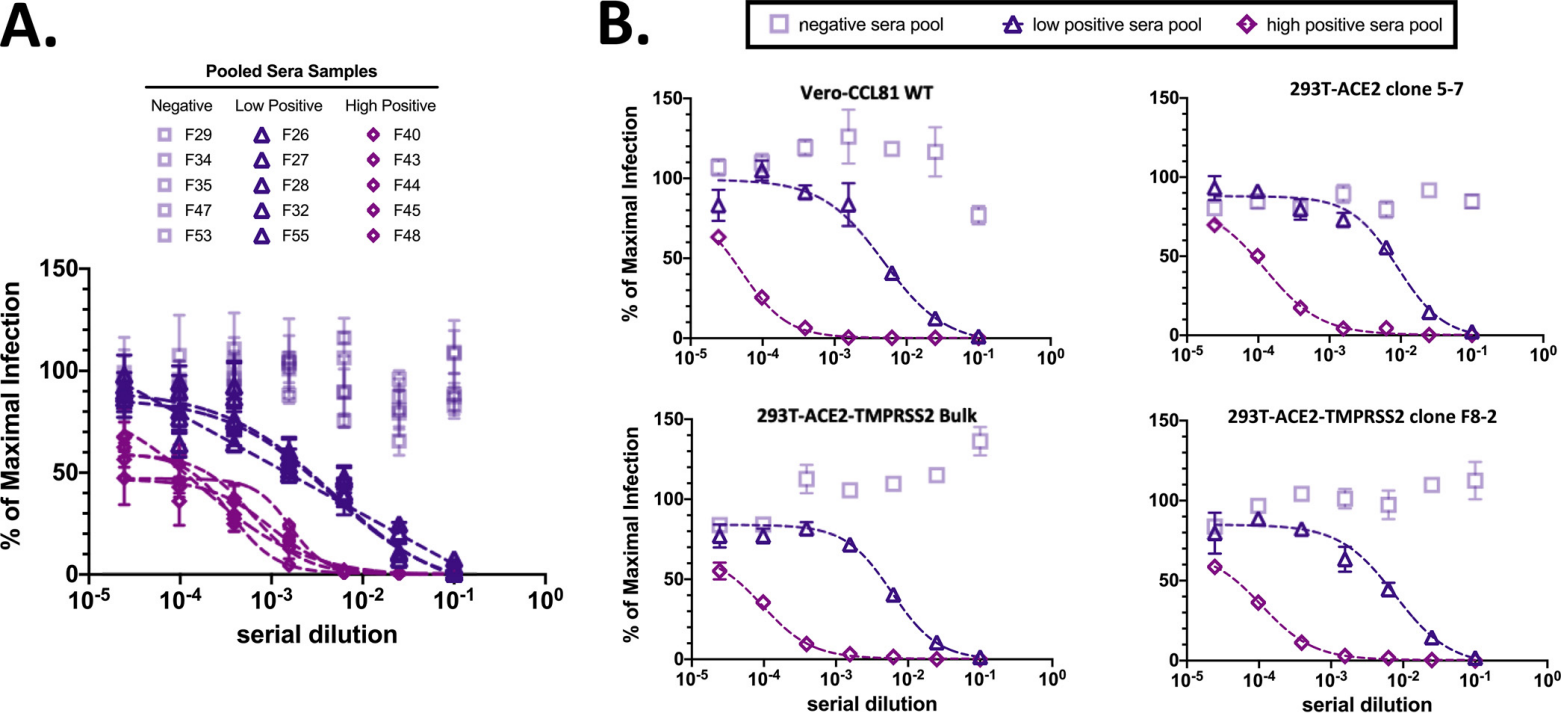
Ultrapermissive 293T-ACE2+TMPRSS2 cell clones retain the same phenotypic sensitivity to convalescent COVID-19 sera. (A) Selection of pooled serum samples. Results from Fig. 4A are reproduced here for the reader’s convenience. Presented is the subset of samples that were pooled for use in VNAs in the adjacent panel. (B) Vero CCL81 and transduced 293T cells were used for VNAs. Sera previously shown to be negative, weakly positive, or strongly positive for CoV2pp neutralization were selected to be pooled in equal volumes. These were subsequently used for VNAs, which were performed and presented as described in the legend of Fig. 4A. Notably, these VNAs were performed in the absence of exogenous trypsin or spinoculation.

We identified serum samples from 15 patients shown in Fig. 4A and placed them into three tiers: negative for CoV2pp neutralization (negative), weakly positive for CoV2pp neutralization (low positive), or strongly positive for CoV2pp neutralization (high positive) (Fig. 7A). We then pooled equal volumes of each set of samples and performed CoV2pp neutralization assays on Vero-CCL81 WT, 293T-ACE2 clone 5-7, and 293T-ACE2+TMPRSS2 bulk-transduced cells and the 293T-ACE2+TMPRSS2 clone F8-2. We demonstrated that even in the case of various levels of ACE2 and TMPRSS2 expression, CoV2pp neutralization assays show consistent patterns of neutralization, exhibiting the robust nature of the assay in tandem with its sensitivity in detecting relative differences in neutralizing titers (Fig. 7B). Patterns of neutralization as well as the calculated absIC_50_ and absIC_80_ reveal a large dynamic range between low- and high-neutralization patient sera across cell lines (Fig. 7B).

## DISCUSSION

Here, we present detailed and optimized protocols for producing VSVΔG-pseudotyped viral particles bearing SARS-CoV-2 spike protein. These CoV2pp recapitulate the SARS-CoV-2 entry requirement for ACE2 expression on the host cell and enhanced infectivity in the presence of activating proteases, such as trypsin and/or TMPRSS2, in both 293T and Vero cells. Evidence from our original standard condition suggested that only a minor fraction of the trypsin added was required, and this trypsin acted at the level of receptor binding on the host cell (Fig. 2C and D). Due to the observed effect of trypsin at the point of infection, we hypothesize that interaction with a cellular factor, likely ACE2, induces conformational changes necessary for further protease-mediated activation, likely at the S2′ cleavage site of SARS-CoV-2 spike. Moreover, in a competitive inhibition assay, entry by the trypsin-treated CoV2pp was successfully inhibited by the sRBD. This faithful recapitulation of the entry processes previously described for SARS-CoV-1 and SARS-CoV-2 suggests that trypsin-treated CoV2pp represent a biologically relevant system for identifying cells that support SARS-CoV-2 entry and for screening for entry inhibitors, especially neutralizing antibodies or patient sera.

Prior to the use of trypsin-treated CoV2pp for neutralization experiments, we assessed how heat inactivation of sera and different cell media affect neutralization. Here, we report detectable neutralization by negative patient sera, which was previously reported for mouse and human sera by Nie et al. (8) However, it is unclear whether the sera used by Nie et al. were heat inactivated. Our observations also raise questions concerning the role that the previously mentioned heat-labile serum factor might play *in vivo*. We have shown that the CoV2pp VNA displays high sensitivity to the inhibition of protease-mediated entry enhancement by human serum, FBS, SBTI, and even nafamostat when the protease in question is TMPRSS2. The inhibitory potential of human serum implies a role that serum factors may play in SARS-CoV-2 pathogenicity, tissue restriction, and systemic spread in previously SARS-CoV-2-naive patients (105). These findings led to the establishment of heat inactivation of sera and use of DMEM plus 10% FBS as conditions for trypsin-treated CoV2pp neutralization experiments. When used for viral neutralization assays with patient sera, the absIC_50_/absIC_80_ against CoV2pp correlated strongly with the results of full-length spike ELISAs and live-virus microneutralization titers. Moreover, we have produced several batches of our CoV2pp and shipped them (along with Vero-CCL81 cells) to many other groups as an out-of-the-box neutralization assay. The first three groups to receive these particles and who volunteered their data have successfully screened patient sera with our assay and observed moderate but significant correlations with the results of spike ELISAs.

While ELISAs provide valuable information about epitopes recognized by individual samples and antibody quantities, functional studies allow for more in-depth analyses of neutralization potential. Notably, RBD-binding antibodies, particularly those inhibiting ACE2, have received a large amount of attention. To identify antibodies capable of preventing receptor binding, many groups have developed competition ELISAs (83), which may serve as a closer surrogate for virus-neutralizing titers. However, recent studies identifying non-RBD binding—yet still neutralizing—antibodies lend insight into novel neutralization mechanisms and further highlight the importance of functional neutralization assays (84, 85). Moreover, our standardized CoV2pp VNA has a large dynamic range that can generate robust neutralization curves, which allows for the calculations of more stringent metrics, such as absIC_50_/absIC_80_ values. absIC_50_/absIC_80_ values give a more meaningful description of the neutralization potential, describing differences that may account for variance in clinical potency (109) of a given serum sample, as many patient sera (and potentially vaccine sera) may not even achieve an absIC_80_. The importance of measuring different thresholds for inhibitory potency has been shown by previous work comparing monoclonal antibodies alone and in combination against different epitopes in order to identify optimal antibody combinations for neutralization (110). Reporting such standardized metrics will allow more meaningful comparisons of vaccine-elicited humoral responses and the neutralization potential of convalescent-phase sera, especially when the latter is used for convalescent-phase plasma therapy. Additionally, we utilized soluble RBD-Fc for neutralizations in two independent labs (Fig. S6) and report comparable values for absolute IC_50_s and IC_80_s. Indeed, the almost-identical IC_80_ values reinforce our proposal that the absIC_80_ is a more accurate and reproducible metric of neutralization potency. The use of standardized metrics and comparisons to standardized samples is of particular importance given the widely varied ratios of spike ELISA binding values and neutralizing antibody titers in comparisons of patients infected by SARS-CoV-2 and patients receiving vaccines for SARS-CoV-2 (13).

10.1128/mBio.02492-20.7 FIG S6 sRBD-Fc potently inhibits CoV2pp entry. Soluble RBD-Fc produced by ISMMS-1 (A) and provided to ISMMS-2 (B). Both groups performed neutralizations of CoV2pp independently and utilized VSV-Gpp as a negative control. Data are presented as normalized to values for an untreated condition (without the sRBD-Fc), and data points were fit using a variable-slope, 4-parameter logistics regression curve (robust fitting method). For ISMMS-1, presented are the neutralizations by SRBD in two independent batches of the sRBD, each performed in technical triplicates. For ISMMS-2, shown are the results from one experiment performed in technical triplicates. Absolute IC_50_ and IC_80_ (i.e., concentrations of the sRBD-Fc) values were calculated as described in Materials and Methods and are presented below the respective graphs. Download FIG S6, TIFF file, 1.2 MB. Copyright © 2021 Oguntuyo et al. 2021 Oguntuyo et al. https://creativecommons.org/licenses/by/4.0/ This content is distributed under the terms of the Creative Commons Attribution 4.0 International license.

Early reports of convalescent-phase serum therapies show a tolerable safety profile and modest benefits from this therapeutic approach (17, 19, 20, 29, 31–33). However, many of these trials consider only ELISA neutralization titers and utilize extremely varied ELISA endpoint titers, from not reported/available to titers ranging from 1:40 to >1:1,350. Interestingly, one pre-peer-reviewed study incorporated functional neutralization studies by utilizing trypsin-treated live virus to screen for sera with >1:80 microneutralization titers on Vero E6 cells (33). Given the wide variance in ELISA titers as well as in virus neutralization titers, we believe that convalescent-phase plasma therapy will be enhanced if patient sera are functionally screened and limited to only those displaying potent neutralization titers. This will have the benefit of transfusing patients only with convalescent-phase sera that have a strong likelihood of substantial *in vivo* inhibitory potential, which is of particular importance given the volumes transfused relative to a patient’s total blood volume. Given our results, a reasonable threshold might be a VNA-derived reciprocal absIC_80_ of ≥343.3 (i.e., ≥75th percentile).

Lastly, we utilize the CoV2pp system to screen 19 single-cell clones and identify two single-cell clones of interest. These clones (293T-ACE2 clone 5-7 and 293T-ACE2+TMPRSS2 clone F8-2) both support effective viral entry in the absence of trypsin and spinoculation and can be used for scaling up viral neutralization assays. The ultrapermissive 293T-ACE2+TMPRSS2 F8-2 clone in particular can support the use of a standardized VNA at the scale needed for screening entry inhibitors, vaccine samples, donor plasma, etc. Our standardized CoV2pp production lot from a single lab, 30 10-cm dishes, was sufficient for ∼12,000 infections/week when the assay was performed in a 96-well format. Trypsin-treated CoV2pp (diluted 1:4) gives a 100:1 signal/noise ratio when performed in a 100-μl infection volume on Vero-CCL81 cells with spinoculation. Using the ultrapermissive F8-2 clone, a 1:50 dilution gives a similar signal/noise ratio without any trypsin treatment or spinoculation. Thus, our weekly production lot becomes sufficient now for ∼150,000 infections/week, which is enough for generating full neutralization curves for ∼4,600 to ∼6,200 samples (assuming an 8-point dilution series performed in quadruplicates or triplicates, respectively). With this, the optimized system utilizes CoV2pp to infect 293T F8-2 clones, which removes the reliance on spinoculation or the addition of exogenous trypsin and will enable the user to have a high dynamic range. Moreover, this system allows for the detection of neutralization by both convalescent-phase plasma and TMPRSS2 inhibition.

Several recently described systems, including VSV encoding the SARS-CoV-2 spike gene (9, 112) and lentiviruses pseudotyped to bear the spike protein (113), are capable of serving as surrogate assays for assessing viral neutralization by patient sera or monoclonal antibodies. The replication-competent VSV system is attractive but still relies on a truncated spike. We have not been able to rescue one with the full-length tail, even in our F8-2 clone, although we could rescue multiple VSVs bearing various betacoronavirus spikes (all with truncated tails). Nonetheless, our standardized CoV2pp based on the VSVΔG system presents many advantages, including safety, ease and speed of use, identity to full-length SARS-CoV-2 spike, versatility for studying spike mutants, and a large dynamic range. First, the viral genome used in this system lacks a viral glycoprotein, which limits the virus to single-cycle replication and mitigates concerns about viral spread. Next, because of the efficient replication of VSV, this system can be used to further interrogate SARS-CoV-2 entry in primary cells and allows for the detection of *Renilla* luciferase (or the desired reporter gene) within 12 to 18 h postinfection. Additionally, the VSVΔG system presented here represents viral entry in the absence of mutations or truncations for enhanced fusogenicity and/or entry dynamics. Lastly, since a viral glycoprotein must be provided in *trans* for every production, this system is not susceptible to mutations over several passages and is not dependent on repeated, arduous rescue attempts for the study of naturally occurring spike mutants or chimeric spike glycoproteins. These studies may prove beneficial as we consider naturally occurring spike mutations—described on platforms such as GISAID—and strive to understand their influence on viral entry kinetics or their influence on escape from antibody neutralization.

In sum, we present detailed and optimized protocols for the production of a BSL2-safe VSVΔG-rLuc pseudoparticle and use it to interrogate viral entry. More importantly, we present several resources that we believe will be invaluable during this global pandemic. This includes cell lines (particularly 293T-ACE2+TMPRSS2 and Vero-CCL81-TMPRSS2 cells) and CoV2pp that are ready to use out of the box for mechanistic studies of viral entry or to screen inhibitors of viral entry. Our findings, resources, and proposed guidelines have implications for standardizing viral neutralization assays, with particular importance for screening therapeutic monoclonal antibodies, vaccine efficacy, and convalescent-phase sera.

## MATERIALS AND METHODS

### Plasmids.

• SARS-CoV-2 spike is in a pCAGG backbone and expresses the codon-optimized Wuhan-Hu-1 isolate (NCBI accession no. NC_045512.2).
• SARS-CoV-2 sRBD (NCBI GenBank accession no. MT380724.1 from the Krammer lab) is in a pCAGG backbone and expresses the codon-optimized sequence from the Wuhan-Hu-1 isolate. The sRBD-His, used for neutralization studies, was generated from this construct.
• VSV-G is in a pCAGG backbone and expresses wild-type Indiana strain VSV-G (Genbank accession no. ACK77583.1).
• ACE2 packaging construct (GeneCopoeia; catalog [cat.] no. EX-U1285-Lv105) uses a cytomegalovirus (CMV) promoter to express ACE2 and bears a puromycin selection marker in the integrating cassette.
• The TMPRSS2 packaging construct (GeneCopoeia; cat. no. EX-Z7591-Lv197) uses a CMV promoter to express TMPRSS2 and bears a blasticidin selection marker in the integrating cassette.
• The psPAX2 2nd-generation lentiviral packaging plasmid (Addgene 12259) expresses HIV-1 Gag, Pol, and Pro proteins.
• The NiV-RBP is in a pCAGG backbone and expresses the hemagglutinin (HA)-tagged, codon-optimized NiV receptor binding protein.

All plasmids listed here are ampicillin resistant. These constructs were transformed into stellar competent cells, grown in bacterial growth media containing carbenicillin, prepared using Invitrogen’s midiprep kit, and sequence verified prior to use for experiments.

### Maintenance and generation of cell lines.

Vero-CCL81 and 293T cells were cultured in DMEM with 10% heat-inactivated FBS at 37°C with 5% CO_2_. VSV-G-pseudotyped lentiviruses packaging ACE2 or TMPRSS2 expression constructs were generated by using Bio-T (Bioland; B01-01) to transfect 293T cells with the second-generation lentiviral packaging plasmid (Addgene; 12259) pCAGG-VSV-G and the desired expression construct (i.e., ACE2 or TMPRSS2). The medium was changed the next morning. Viral supernatant was collected 48 h posttransfection, clarified by centrifugation at 4,000 rpm for 5 min, and aliquoted prior to storage at −80°C. Vero-CCL81 and 293T cells were transduced in a 6-well plate with the prepared lentiviral constructs. Two days after transduction, these cells were expanded into a 10-cm plate and placed under selection with puromycin (for ACE2-transduced cells) or blasticidin (for TMPRSS2-transduced cells). 293T and Vero-CCL81 cells were selected with 2 and 10 μg/ml of puromycin, respectively. For blasticidin, 293T cells were selected with 5 μg/ml and Vero-CCL81 cells were selected with 15 μg/ml. To generate ACE2- and TMPRSS2-expressing 293T cells, 293T-ACE2 cells were transduced with VSV-G-pseudotyped lentivirus-packaged TMPRSS2. These cells were subsequently selected with 5 μg/ml blasticidin. Low-passage-number stocks of each cell line generated were immediately frozen down using Bambanker (Fisher Scientific; NC9582225). Single-cell, isogenic clones were isolated via serial dilution in a 96-well plate. Wells with only a single cell were grown up and eventually expanded while under selection.

### Pseudovirus production and titering.

We provide detailed production and titering protocols in the supplementary text (Text S1). Briefly, 293T producer cells were transfected to overexpress SARS-CoV-2 or VSV-G glycoproteins. For background entry with particles lacking a viral surface glycoprotein, the pCAGG empty vector was transfected into 293T cells. Approximately 8 h posttransfection, cells were infected with the VSVΔG-rLuc reporter virus for 2 h and then washed with Dulbecco’s phosphate-buffered saline (DPBS). Two days postinfection, supernatants were collected and clarified by centrifugation at 1,250 rpm for 5 min. For trypsin-treated CoV2pp, a small batch of particles was first treated with TPCK-treated trypsin (Sigma-Aldrich; T1426-1G) at room temperature for 15 min prior to inhibition with soybean trypsin inhibitor (SBTI) (Fisher Scientific; 17075029). Subsequently, the rest of the CoV2pp were trypsin treated. All particles were aliquoted prior to storage at −80°C to avoid multiple freeze-thaws.

To titer these pseudoviruses, 20,000 Vero-CCL81 cells were seeded in a 96-well plate 20 to 24 h prior to infection. Single aliquots of BALDpp, CoV2pp, and VSV-Gpp were used for infections, and titrations were performed in technical triplicates. At 18 to 22 h postinfection, the infected cells were washed with DPBS, lysed with passive lysis buffer, and processed for detection of *Renilla* luciferase. A Cytation3 apparatus (BioTek) was used to read luminescence. Additional details can be found in Text S1.

### Collection of producer cells and concentration of pseudotyped particles.

Cell lysates were collected from producer cells with 10 mM EDTA in DPBS. Cells were subsequently lysed with radioimmunoprecipitation assay (RIPA) buffer (Thermo Scientific; 89900) containing protease inhibitor (Thermo Scientific; 87785) for 30 min on ice. Lysates were centrifuged at 25,000 × *g* for 30 min at 4°C, and the supernatants were collected and stored at −80°C. Total protein concentrations were determined by the Bradford assay. For viral pseudoparticles, 10 ml of designated viral particles was concentrated via a 20% sucrose cushion (20% sucrose in DPBS), an Amicon Ultra centrifugal filter (100-kDa cutoff, UFC910024; Millipore Sigma), or polyethylene glycol (PEG) precipitation (Abcam; ab102538). Concentrated viral particles were resuspended in 300 μl of PBS or Opti-MEM for further analysis.

### Western blots.

All protein samples were run under reduced conditions by dilution in 6× SDS containing dithiothreitol (DTT) and 5% beta-mercaptoethanol (Fisher Scientific; ICN19483425). The protein was subsequently incubated in a heating block at 95°C for 15 min, run on a 4 to 15% SDS-PAGE gel, and transferred to polyvinylidene difluoride (PVDF) membranes (Bio-Rad). Membranes were blocked with phosphate-buffered saline blocking buffer (LI-COR; 927-700001) and then probed with the following antibodies. Antibodies against SARS-CoV2 (2B3E5 from Thomas Moran and GTX632604 from GeneTex), ACE2 (66699-1-Ig from Proteintech and Rb ab108252 from Abcam), VSV-G (A00199 from GenScript), VSV-M (EB0011 from Kerafast), anti-HA (NB600-363 from Novus), and CoX IV (926-42214 from LI-COR) were used. For secondary staining, membranes were washed and incubated with the appropriate Alexa Fluor 647-conjugated anti-mouse antibody or Alexa Fluor 647-conjugated anti-rabbit antibody. Alexa Fluor 647 was detected using the ChemiDoc MP imaging system (Bio-Rad). Relative ACE2 or TMPRSS2 abundance was calculated by first normalizing abundance relative to GAPDH (glyceraldehyde-3-phosphate dehydrogenase) expression and then normalization to wild-type expression.

### RNA extraction and qPCR for ACE2 and TMPRSS2 expression.

Total RNA was extracted from cells using a Direct-zol RNA miniprep kit (Zymo Research; R2051), and reverse transcription (RT) was performed with the Tetro cDNA synthesis kit (Bioline; BIO-65043) and random hexamers. RT-PCR was performed with the SensiFAST SYBR and fluorescein kit (Bioline; BIO-96005). For qPCRs, hypoxanthine phosphoribosyltransferase (HPRT) forward (5′-ATTGTAATGACCAGTCAACAGGG-3′) and reverse (5′-GCATTGTTTTGCCAGTGTCAA-3′) primers, ACE2 forward (5′-GGCCGAGAAGTTCTTTGTATCT-3′) and reverse (5′-CCCAACTATCTCTCGCTTCATC-3′) primers, and TMPRSS2 forward (5′-CCATGGATACCAACCGGAAA-3′) and reverse (5′-GGATGAAGTTTGGTCCGTAGAG-3′) primers were utilized. Samples were read on the CFX96 Touch real-time PCR detection system (Bio-Rad). For qPCR, forward and reverse primers were utilized. The qPCR was performed in duplicates for each sample, and results were calculated using 2^–ΔΔ^*^CT^*, where *CT* is threshold cycle, with normalization to the HPRT housekeeping gene control and further normalization to the 293T parental cells.

### Serum acquisition.

All patient sera were acquired after approval by the respective institutional review boards (IRBs) and/or equivalent oversight bodies (Bioethics Committee, Independent Ethics Committee), as follows: (i) the Mount Sinai Hospital IRB (New York, NY, USA), (ii) the Louisiana State University Health Sciences Center—Shreveport (LSUHS, LA, USA), and (iii) the Fundacion Instituto Leloir-CONICET, Universidad Nacional de San Martin, Laboratorio Lemos SRL, Universidad de Buenos Aires (COVIDAR Argentina Consortium, Buenos Aires, Argentina). Samples were deidentified at the source institutions or by the respective principal investigators (PIs) of the IRB-approved protocols for sample collection before analyses performed in this study. All necessary patient/participant consent has been obtained, and the appropriate institutional forms have been archived.

### ELISAs and live-virus neutralization.

Spike ELISAs for patient sera from the Krammer lab were performed in a clinical setting using the two-step protocol previously published (Mount Sinai Hospital). Briefly, this involves screening patient sera (at a 1:50 dilution) with the sRBD; samples determined to be positive were further screened at 5 dilutions for reactivity to the spike ectodomain. All 36 samples were screened in this manner, but a subset of 15 samples were further screened for IgG and IgM antibodies binding to the spike ectodomain. The protocol from Stadlbauer et al. (106) was modified slightly to start from a 1:300 and end at a 1:24,300 dilution of serum. IgG and IgM antibodies were detected with secondary antibodies conjugated to horseradish peroxidase (HRP) (Millipore AP101P for anti-human IgG and Invitrogen A18841 for anti-human IgM). Background was subtracted from the OD values, samples were determined to be positive if their ODs were ≥3-fold over that of the negative control, and the AUC was calculated in PRISM. ELISAs performed by the LSUHS group utilized the sRBD with a 1:50 dilution of patient serum to screen all samples, followed by use of the spike ectodomain with patient sera at a 1:100 dilution. Background-subtracted OD values are reported for both sets of ELISAs. ELISAs performed by the COVIDAR group utilized a mixture of the sRBD and the spike ectodomain for samples serially diluted from 1:50 to 1:6,400. AUCs were calculated as described above.

All live-virus neutralizations were performed at biosafety level 3 (BSL3) using the USA-WA/2020 isolate of SARS-CoV-2 as described by Amanat et al. (73). Briefly, ∼600 50% tissue culture infective doses (TCID_50_s) of virus were incubated with a serial dilution of patient sera for 1 h at 37°C prior to infection of Vero-E6 cells. Forty-eight hours postinfection, cells were fixed in 10% paraformaldehyde (PFA) and stained with mouse anti-SARS-CoV nucleoprotein antibody. This was subsequently detected by the addition of HRP-conjugated goat anti-mouse IgG and Sigmafast OPD. The BioTek Synergy 4 plate reader was used to measure the OD at 490 nm (OD_490_), which was subsequently used to calculate microneutralization (MN) titers. The samples with live-virus MN titers were a part of a larger study by Krammer and colleagues looking at the longitudinal dynamics of the humoral immune response. This study was recently posted on medRxiv (35). We obtained permission from the authors to utilize a random subset of serum samples from their study and their associated MN titers for validation studies with our CoV2pp-based virus neutralization assay (VNA).

### Neutralization studies with patient sera, the soluble RBD, or nafamostat-mesylate.

Deidentified sera were obtained with IRB approval to use for research purposes. Unless otherwise noted, all patient sera were heat inactivated at 56°C for 30 min and serially diluted in DMEM plus 10% FCS when we performed VNAs. For groups receiving our CoV2pp, we recommended titrating our stocks first to determine the linear dynamic range that would be useful for VNAs done in their labs. As a quality control, we send out only CoV2pp stocks that give signal/noise ratios of at least 100-fold over that of BALDpp when diluted 4-fold in a 100-μl total infection volume in a 96-well plate format. For the VNAs performed in our lab (ISMMS-1), a pretitrated amount of pseudotyped particles (diluted to give approximately 10^5^ RLUs) was incubated with a 4-fold serial dilution of patient sera for 30 min at room temperature prior to infection of Vero-CCL81 cells seeded the previous day. For sRBD or nafamostat inhibition, a pretitrated amount of pseudotyped particle dilution was mixed with the protein or compound and added to cells immediately after. Approximately 20 h postinfection, cells were processed for the detection of luciferase activity as described above. Our recommendations to generate a robust neutralization curve were to do an 8-point serial dilution curve, with each point done in triplicate. Raw luminometry data were obtained from labs that volunteered VNA results from at least 12 patient samples and analyzed as indicated below.

Method modifications from the three contributing labs are as follows. Serum neutralizations by LSUHS (J. P. Kamil and S. S. Ivanov) were performed by first diluting the serum 4-fold in a 100-μl total volume and then diluting it via a 3-fold serial dilution. Cell lysates were transferred to a white-walled 96-well plate, and then the Promega *Renilla* luciferase assay kit was utilized to detect luciferase. Plates were read on a Tecan Spark plate reader by collecting total luminescence signal for 10 s. ISMMS-2 (C. E. Hioe) began neutralizations at a 10-fold dilution and proceeded with a 4-fold serial dilution. Plates were read on a black-walled 96-well plate using the *Renilla* Glo substrate (Promega; E2720), with a 1-s signal integration time. COVID-19 samples were provided to ISMMS-2 by the Clinical Pathology Laboratory at ISMMS or from an IRB-approved study at the James J. Peters VA Medical Center. COVIDAR (A. V. Gamarnik) began at either an 8-fold or a 16-fold dilution and then continued with either a 3-fold or a 2-fold serial dilution, respectively. White, F-bottom Lumitrac plates (Greiner; 655074) were read via the GloMax Navigator microplate luminometer (Promega; GM200) using the ONE-Glo luciferase assay system (Promega; E6110).

### IC calculations and other R packages used.

Relative inhibitory concentration (IC) values were calculated for all patient serum samples by modeling a 4-parameter logistic regression with drm in the R drc package (114). For examples, a relative inhibitory concentration of 50% (IC_50_) is calculated as the midway point between the upper and lower plateaus of the curve. The absolute inhibitory concentration (absIC) was calculated as the corresponding point between the 0% and 100% assay controls. For example, the absIC_50_ is the point at which the curve matches inhibition equal to exactly 50% of the 100% assay control relative to the assay minimum (0%) (115). As a result, serum samples that are nonneutralizing or minimally neutralizing may have lower plateaus, indicating that they cannot reach certain absolute inhibitory concentrations, such as an absIC_90_ or absIC_99_. R was also used to generate the heatmaps presented in Fig. 4B and Fig. 5A as well as the plots in Fig. 4D and 5C and Fig. S7.
