# Supplementary material for: Quantifying Absolute Neutralization Titers against SARS-CoV-2 by a Standardized Virus Neutralization Assay Allows for Cross-Cohort Comparisons of COVID-19 Sera
Source: mBio. 2021 Feb 16;12(1):e02492-20. doi: 10.1128/mBio.02492-20 (PMC8545089; doi:10.1128/mBio.02492-20)
Supplement: TEXT S1 [file mbio.02492-20-stext01.docx]

**Production of SARS-CoV-2 VSV-ΔG rLuc Pseudotyped particles* (last updated December 2020) *modified from HNVpp protocol**

Production of VSV-ΔG rLuc Pseudoparticles

**Purpose:** The following procedure should be used to generate VSV-ΔG particles pseudotyped with CoV-2 Spike (S) glycoprotein. These purified stocks can then be used to perform single-round infection in various cell types using BSL-2 conditions.

**Biosafety:** While all virus generated through this protocol is bio-contained by being limited to single-cycle infection, all aspects of this protocol should be carried out with standard sterile tissue culture technique, and BSL-2 precautions whenever handling VSV. See more information below for more detailed biosafety considerations.

Our VSV∆G reporter backbone is from the Indiana lab-adapted strain which is cleared for use at BSL-2 (reviewed in PMID: [20709108](https://pubmed.ncbi.nlm.nih.gov/20709108/)). Seed stocks of VSV∆G trans-complemented with VSV-G have been used all over the world for more than 20 years with no reports of recombinant replication-competent VSV being generated. During the course of this pandemic, our colleagues in the U.K., Australia, and Taiwan have received and obtained permission to import our VSV∆G CoV2pp for use in their countries. Furthermore, *bona fide* recombination amongst negative sense RNA viruses (as opposed to positive sense RNA viruses) is exceedingly rare if not absent (reviewed in PMID: [21994784](https://www.ncbi.nlm.nih.gov/pubmed/21994784)). Finally, our VSV-G provided *in trans* lacks the VSV-G gene start and gene stop signals present in the VSV∆G backbone, making even the possibility of a productive homologous recombination event vanishingly small.

**Materials needed:**

- Corning Falcon standard 10 cm TC dishes cat no. 08-772E
- Sigma Poly-L-Lysine solution, 0.01% m/w cat no. 4707
- Kerafast anti-VSV-G [8G5F11] antibody cat no. EB0010
- Polysciences Transporter 5 (PEI) Transfection Reagent cat no. 26008-5
- Promega Renilla Luciferase Assay System cat no. E2820
- sterile ddH2O
- sterile DPBS
- 2, 5, 10, and 25mL sterile pipettes
- barrier p2 through 1000 pipette tips
- p2 through 1000 micropipettors and p200 multichannel
- Falcon 96 well flat bottom plate for Tissue culture cat no. 08-772-3
- Alkali Scientific 96 well V bottom for virus titration cat no. TL598
- VSV[Rluc]- ΔG-G* parental stock, stored at -80º C and with known titer
- sterile midi or maxiprep of surface glycoprotein(s) expression plasmid
- If concentrating supernatant by ultracentrifugation:
  - Seton Scientific open-top polyclear tubes (38.5 mL capacity) cat no. NC9115402
  - Balance with sensitivity to at least 0.01 g
  - 20% w/v sucrose in DPBS + 1mM EDTA (sterilized through 0.22um filter)
- If trypsin treating particles: TPCK Trypsin and Soybean Trypsin Inhibitor

**Procedure:**

I. Transfection of 293T cells in 10 cm plates with surface glycoprotein construct:

1. If not using collagen coated plates, before seeding, coat 10cm dishes with poly-L-lysine (PLL)*:
   1. Dilute PLL stock 1:10 with sterile ddH2O
   2. Add approx. 5mL PLL dilution to each plate, then rotate and tilt plates until all surfaces are coated with liquid
   3. Incubate for >15min in 37ºC incubator
   4. Remove PLL, wash plates with sterile DPBS and aspirate until dry

*Collagen coated plates, though more expensive, keep the cells more adherent and healthier through the wash steps and media exchanges.

1. At ~20 hours before transfection, plate 6 X 10^6^ 293T cells in PLL-coated 10 cm dishes.
2. At time 0h (~20hrs post-seeding), replace the media with 5mL DMEM+10% FBS^i^, then transfect 24 μg total DNA^ii^ (glycoprotein expression plasmid) per plate:
3. Dilute 120uL PEI reagent in 500uL PBS
4. Dilute total 24ug of DNA in a separate tube of 500uL PBS
5. Add PEI mix to DNA mix and incubate at room temperature for 30mins.
6. Add mixture dropwise to cells.
7. The purpose of this media exchange is to reduce the volume that the transfection reagents are added to i.e. 5mLs instead of 10mLs.
8. For viruses requiring only a single-entry glycoprotein, use up to 12µg of that plasmid along with 12µg of empty vector to bring total DNA concentration up to 24µg total. For viruses requiring multiple entry glycoproteins, use equal amounts of each glycoprotein e.g. Henipaviruses require a fusion and attachment glycoprotein so 12µg of F and 12µg of RBP are transfected into the 293T cells. However, this is something that may need further optimization for your purposes.

II. Infection of transfected cells ~8 hours* post transfection:

*The timing of VSV-ΔG-G* infection depends on the cell surface expression kinetics of your envelope protein, we recommend optimizing with 6, 8, 12, and 24 hpt to determine ideal timing.

1. At ~8hpt, infect with a VSV[Rluc]- ΔG-G* stock (~1 X 10^8^ TCID_50_ units) in an inoculum volume of 5 mL per plate.
2. Incubate inoculum for 1-2 hours at 37^o^ C to permit infection
3. Remove inoculum
4. Wash plates 3X with dPBS to remove excess VSV-G particles that did not infect. Be extremely careful when pipetting dPBS on/off the 293Ts to avoid detachment.
5. Prepare incubation medium, optiMEM* containing a 1:20,000 dilution of anti-VSV-G antibody (8G5F11 from Kerafast).**

*We currently use optiMEM for CoV2pp production since we observed an increase of CoV-2 spike cleavage relative to DMEM+10%FBS.

**We find that this potently neutralizing antibody minimizes the background sometimes seen with “bald” VSV pseudotypes (293T cells transfected with 24µg empty vector, then infected as normal). If making VSV-Gpp in parallel, take care not to incubate that plate with anti-VSV-G containing media.

1. Add 10mL incubation medium to each plate of washed 293T cells
2. At 48 hpi collect supernatant into a 50 mL conical tube — roughly 10 mL per plate

III. Purification of pseudoparticles by clarification and ultracentrifugation* (CoV2pp):

*CoV2pp are currently clarified, but not concentrated through a sucrose cushion. If needed, other concentration methods to consider are Amicon and Peg concentration. All methods work well for concentrating particles prior to using for western blot.

1. Clarify the pseudotype-containing supernatant of cell debris by centrifugation in a standard benchtop centrifuge at 1,250 rpm (approximately. 450xg) for 5 min
2. Transfer clarified supernatant to Seton Open-Top Polyclear Centrifuge Tubes for SW28 rotor. Add 15 mL serum-free media.
3. Insert pipette containing 10.5mL 20% surcrose to the bottom of the tube and gently add 10 mL 20% sucrose to the bottom of the tube to create cushion. Once done, carefully remove pipette without disrupting the sucrose-media interface.
4. Generate a balance tube of identical mass – within 0.2g – using plain DMEM and 20% sucrose. Balance with plain DMEM.
5. Ultra-centrifuge with SW28 rotor and buckets for 2 hours at 25,000 rpm at 4 ^o^C.
6. Pour off supernatant into bleach; use filtered 2mL pipette to remove remaining beads of liquid from the tube
7. Resuspend pellet of concentrated pseudoparticles in 600 μL DPBS
8. Make four 110 μL aliquots for experiments and one ~200 uL aliquot for TCID_50_ titration (part IV).

IV. Titrate by limiting dilution on susceptible cell line:

1. ~24h prior to infection, seed ~20,000 Vero cells per well in a 96-well plate.
2. At time 0h, prepare serial 1:10 dilutions of your virus stock in your desired media (i.e. DMEM+10%FBS for Vero cells) such that you are able to transfer a final volume of 100uL inoculum per well. Perform this dilution series in 6 replicates in order to generate a TCID_50_ value.
3. At 18-22 hpi:
   1. Prepare Promega Renilla or Passive Lysis buffer by diluting 1/5 in ddH2O
   2. Remove culture media/inoculum
   3. Wash each well with 100µL of DPBS, then removing this with a multichannel
   4. Lyse* cells by adding 25µL prepared lysis buffer to each well. From this point on, all samples can be handled safely outside the biosafety cabinet, as all living and infectious material has been inactivated.

*It is recommended that, in addition to using the passive lysis buffer provided in the kit, one freeze-thaw cycle is performed to release the rLuc. An orbital shaker for 15mins at 500rpm is also a viable option.

- 1. After lysis the same plate is assayed for Renilla luciferase* production on a plate reader using the Promega rLuc kit.

*For preparing the Renilla luciferase assay reagent, we dilute the assay buffer 1:1 with DPBS and use a 1:200 dilution of assay substrate. For example, when producing a total of 10mL of assay reagent, we use 5mL of DPBS with 5mL of assay buffer and 50µL of assay substrate.

When reading on the Cytation3, we use this procedure: (1) delay of 5 seconds between each well, (2) dispense 40µL of assay reagent, (3) shake for 2 seconds, (4) delay for 2 seconds, (5) read luminescence, (6) quench the reaction by dispensing 50µL of 70% ethanol, (7), shake for 5 seconds and (8) proceed to the next well.

1. The limit of detection for the Cytation3 is ~300 RLUs. When calculating TCID_50_, we use the Spearman & Karber algorithm. Positive wells are those with >2x the average background signal i.e. for the Cytation3, this would be >600 RLUs.
   - For other instruments, assay uninfected wells to determine the background signal.

V. TPCK Trypsin treat CoV2pp to enhance entry:

1. Once a titer has been established for this batch (or in parallel with titering the untreated stock) of CoV2pp, we strongly recommend immediately treating a small batch (~400-500µL) of CoV2pp with a serial dilution of TPCK trypsin and one concentration of soybean inhibitor. This will establish what concentrations should be used for treating your larger batch of particles prior to storage at -80ºC.
   1. Trypsin treatment should be as follows:
      1. Add the desired concentration of trypsin and incubate at room temperature for 15mins
      2. Add the desired concentration of soybean inhibitor, then store at -80ºC
      3. Thaw one tube for titering
   2. For two separate batches of trypsin, we have had success with the following conditions:
      1. 1^st^ batch: 625µg/mL of trypsin with 625µg/mL of soybean inhibitor
      2. 2^nd^ batch: 475µg/mL of trypsin with 600µg/mL of soybean inhibitor
      3. From further testing of trypsin treatments, we hypothesize that despite inactivation with soybean inhibitor, there is some “leaky” trypsin that retains activity until it interacts with cells to enhance/prime SARS-CoV-2 spike mediated infection. We also observe that there is an extremely narrow window (likely between 6.25µg/mL and 12.5µg/mL) for trypsin to induce this entry enhancement. Taken together, we speculate that drastically lower concentrations of trypsin and soybean inhibitor can be used to achieve maximal CoV2pp entry with limited toxicity.
   3. General notes:
   4. While establishing a titer for the CoV2pp produced, we only recommend storing the particles in 4ºC for up to two days.
   5. We also recommend producing ample stock of TPCK trypsin and soybean inhibitor to be stored in -20ºC. Do not re-freeze and thaw tubes of trypsin or soybean inhibitor. This will limit repeatedly having to repeatedly test the optimal concentrations that limit toxicity while maximizing CoV2pp entry.
2. After deciding on the optimal trypsin condition, trypsin treat the remaining batch, aliquot appropriately and store at -80ºC

**Storage and Stopping**

- All supernatants must be stored at -80º C
- Stocks can be frozen prior to titration, but not prior to purification through sucrose
- Titration plates can be frozen at -20C (up to 1wk) or -80º C (flash-freeze, or long-term storage) once they are in lysis buffer

**Expected Timeline**

Day -1 (pre-infection)

- PLL coat 10cm dishes
- Seed 293T cells

Day 0: transfect and infect 293T cells

- Transfect 293T cells
- Infect transfected cells with parental VSV[Rluc]- ΔG-G* stock

Day 1 post-infection

- Seed susceptible cells in 96-well plates for titration (optional)

Day 2 post-infection

- Collect supernatant from transfected/infected cells
- Clarify and, if needed, concentrate (i.e ultracentrifugation, Amicon filter, or PEG) supernatant
- If needed, trypsin-treat CoV2pp, aliquot and freeze
- Titer new pseudotyped virus stocks on susceptible cells

Day 3 post-infection

- Assay Renilla luciferase for titer of pseudotyped stocks


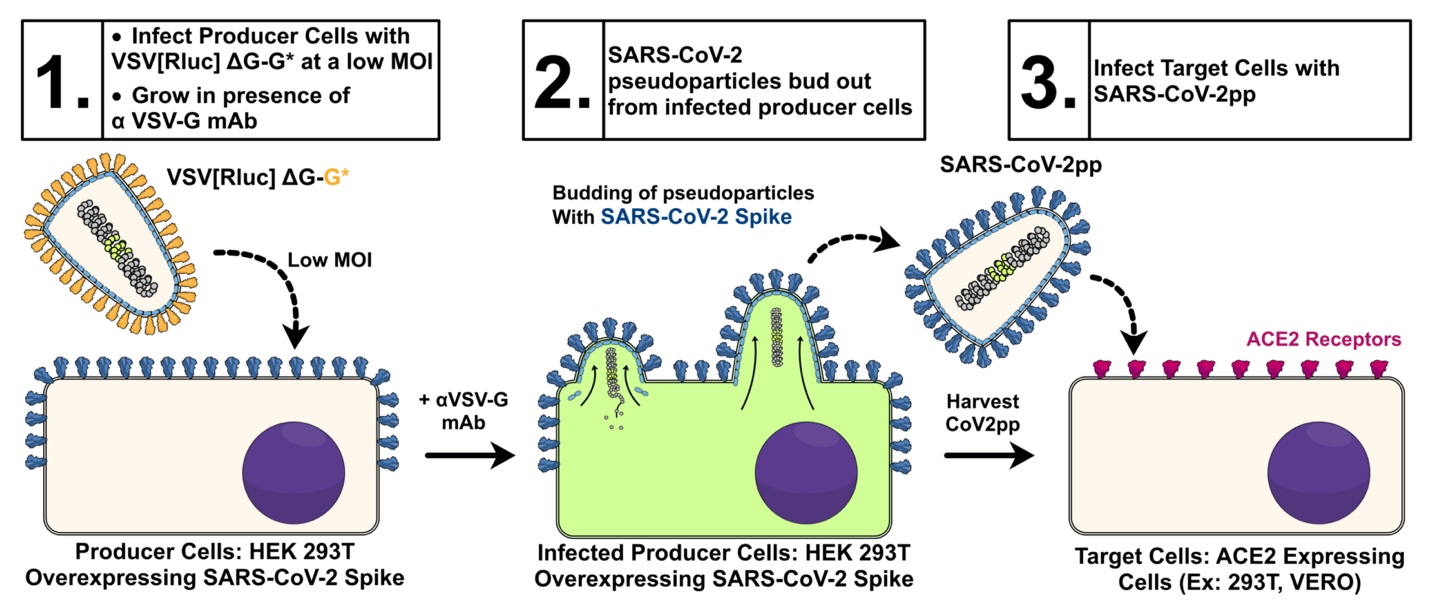


**Protocol for titering CoV2pp and for neutralization studies**

**Part I: Protocol for Tittering CoV2pp by limiting dilution on susceptible cell lines**

**Part II: Protocol for neutralization studies with monoclonal antibodies or patient sera.**

**Materials needed:**

- Cells/culture media:
  - Vero CCL81 cells or isogenic cells (293T-ACE2 clone 5-7 or 293T-ACE2+TMPRSS2 clone F8), all maintained in DMEM+10% FBS
  - DMEM with 10% FBS
  - Sterile DPBS
  - Sterile ddH20
- General lab equipment and consumables:
  - Barrier p100 through 1000 sterile pipette tips
  - P100 through 1000 micropipettors and p200 multichannel
  - 96 well flat bottom plate for tissue culture
  - 96 well V bottom for serum titrations
  - Sterile and non-sterile reagent reservoirs/basins
  - Tabletop centrifuge (with 96 well plate holders)
  - Cell culture incubator
  - Bleach
  - 70% ethanol
- Assay specific reagents/tools:
  - Luminescence plate reader (we use BioTek’s Cytation3)
  - Promega *Renilla* luciferase assay system (100 assays-E2810; 1000 assays-E2820)
  - CoV2pp: VSV∆G-Rluc bearing SARS-CoV-2 Spike glycoprotein
  - VSV-Gpp: VSV∆G-Rluc bearing VSV-G entry glycoprotein
  - BALDpp: VSV-G-Rluc bearing no protein (produced in parallel with samples above)

**Safety notes:**

- The CoV2pp are a VSV pseudotyped particle (pp) system that do not encode any viral glycoprotein in the VSV genome and can be worked with under Bio-Safety Level 2 (BSL2) conditions. Regardless, out of an abundance of caution, we recommend taking the appropriate precautions for the user’s safety. During and after completing an experiment all tips or surfaces that come in contact with the CoV2pp are brought into contact with a bleach solution. To achieve this, we bring a 20% bleach solution into the tissue culture (TC) hood, which allows us to add up to the equivalent volume while still keeping the bleach concentration ≥10%. For example, bringing in 200mL of a 20% bleach solution into the TC hood allows for 200mL of extra fluids to be inactivated by this solution. Upon the completion of experiments, the bleach bucket should be left in the hood for ≥15 minutes prior to removing it and disposing of the inactivated solution (i.e. down the sink drain). Alternatively, quatracide, is a potent, inactivating substance that should be used according to the manufacturer’s instructions.

1. **Protocol for Titering CoV2pp by limiting dilution on susceptible cell lines:**
2. ~24 hours prior to infection, seed 20,000 Vero CCL81 cells* per well in a 96-well plate
   1. Grown in standard DMEM+10% FBS

*if using 293T clone 5-7 or F8 cell lines, seed ~35,000 cells per well

1. At time 0hrs, prepare your desired serial dilution of virus in Serum Free Media (SFM; DMEM only) such that you are able to transfer a final volume of 100µL/well*

*We recommend performing this in triplicate in order to generate a TCID_50_ value

- 1. For example, for a 5-fold serial dilution:
     1. add 120µL SFM to all wells except the top well
     2. Add 200µL of undiluted CoV2pp to the top well
     3. For a fivefold serial dilution, transfer 30µL of the previous dilution to subsequent wells
     4. Carefully remove the media from your Vero cells
     5. Starting from the lowest dilution, carefully transfer 100µL from your titration plate to your cells
  2. Move plates to 37ºC incubator

1. At 18-22hrs post infection:
   1. Prepare the lysis buffer
      1. For the Promega Renilla lysis buffer, dilute 1:5 in ddH_2_O for 1X
   2. Remove your culture media/inoculum
   3. Wash each well by adding 100µL of DPBS, then removing this volume with a multichannel
   4. Lyse* cells by adding 25µL of prepared lysis buffer to each well

*It is recommended that, in addition to using the passive lysis buffer provided in the kit, one freeze-thaw cycle or vigorous shaking on an orbital shaker (i.e. 500rpm for 15mins) is performed to release the rLuc.

- 1. After lysis and one freeze thaw, the same plate is assayed for Renilla luciferase* production on a plate reader using the promega rLuc kit

* For preparing the Renilla luciferase assay reagent, we dilute the assay buffer 1:1 with DPBS and use a 1:200 dilution of assay substrate. For example, when producing a total of 10mL of assay reagent, we use 5mL of DPBS with 5mL of assay buffer and 50µL of assay substrate.

When reading on the Cytation3, we use this procedure: (1) delay of 5 seconds between each well, (2) dispense 40µL of assay reagent, (3) shake for 2 seconds, (4) delay for 2 seconds, (5) read luminescence, (6) quench the reaction by dispensing 50µL of 70% ethanol, (7) shake for 5 seconds and (8) proceed to the next well.

1. Calculating TCID50, we use the Spearman & Karber algorithm. Positive wells are those with >3x the average background signal of uninfected wells.*

*For the Cytation3, the limit of detection is 300 RLUs. When calculating TCID50, we consider positive wells as those with >1000 RLUs. For other instruments, we recommend assaying multiple uninfected wells to determine the background signal.

1. **Protocol for neutralization studies with monoclonal antibodies or patient sera:**
   1. 24 hours prior to infection, seed 20,000 Vero CCL81 cells per well in a 96-well plate
   2. At time 0hrs, prepare a dilution of your monoclonal antibody or sera in DMEM+10%FBS in a V bottom 96 well plate:
      1. If using a 4-fold serial dilution, add 28.75µL media to all wells except the top well. To the top well, add 38.34µL of your entry inhibitor to the well containing your starting dilution, then transfer 9.58µL for a 4-fold serial dilution. This ensures that there is 28.75µL of your entry inhibitor (e.g. sera, monoclonal antibody or small molecule) in each well, which will be further diluted 1:4 after the addition of virus.
   3. Dilute your virus stock in DMEM+10%FBS* and, using a multichannel and a sterile basin, transfer the desired amount of virus to each well of a V bottom 96 well plate
      1. If using a 4-fold serial dilution of your entry inhibitor, transfer 86.25µL of virus to each well. This further dilutes your entry inhibitor 4x. For patient sera, we recommend a final dilution range of 1:10 to 1:40,960, which covers a wide neutralization range..

*Based on your needs and the cell lines being utilized for entry inhibition studies, we recommend between a 1:4 and 1:50 dilution of the CoV2pp. In our hands, when using Vero-CCL81 cells, RLUs ~10^5^ (>100x signal:noise) are achieved with a 1:4 dilution of CoV2pp. Whereas, when using either the 293T-ACE2 clone 5-7 or 293T-ACE2-TMPRSS2 clone F-8, the same RLUs can be achieved with a 1:20-1:50 dilution. Regardless, we strongly recommend titering all CoV2pp batches first prior to use for viral neutralization assays or entry inhibition assays. We recommend performing each point of the neutralization curve in triplicate. Each time the experiment is performed, we also recommend having at least one positive and negative control mAb. See the plate layout below.

- 1. Remove media from your cells.
  2. Starting from the lowest serial dilution, transfer 100µL of your mix to the cells
  3. Move plates to 37ºC incubator
  4. After 18-22hrs, lyse and read plates as described in Part I3
     1. Briefly, lyse plates with 25µL of 1X lysis buffer. Freeze plate in -80ºC, then thaw for one freeze thaw cycle. After reaching room temperature, read plates with a luminescence plate reader like the Cytation3.


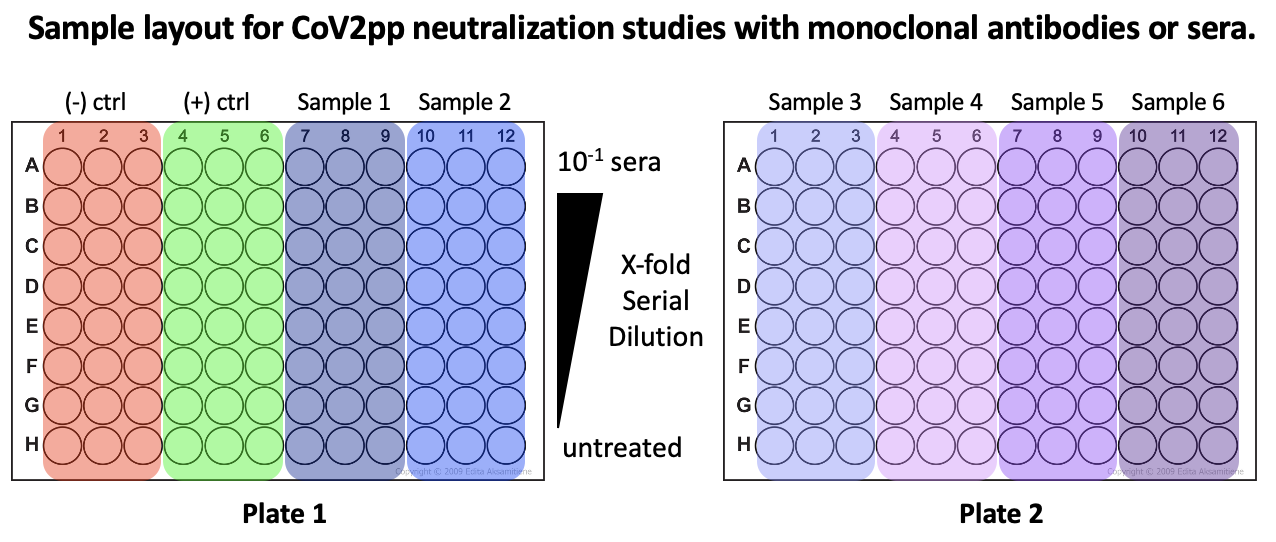


**Storage and Stopping:**

- All CoV2pp should be stored at -80ºC until needed. We do not recommend additional freeze-thaws of CoV2pp as that will likely result in reduced titers.
- Once in lysis buffer, plates can be frozen at -20ºC (for up to 1 week) or at -80ºC (for longer term storage).
