# Supplementary figures and images for: Quantifying Absolute Neutralization Titers against SARS-CoV-2 by a Standardized Virus Neutralization Assay Allows for Cross-Cohort Comparisons of COVID-19 Sera
Source: mBio. 2021 Feb 16;12(1):e02492-20. doi: 10.1128/mBio.02492-20 (PMC8545089; doi:10.1128/mBio.02492-20)

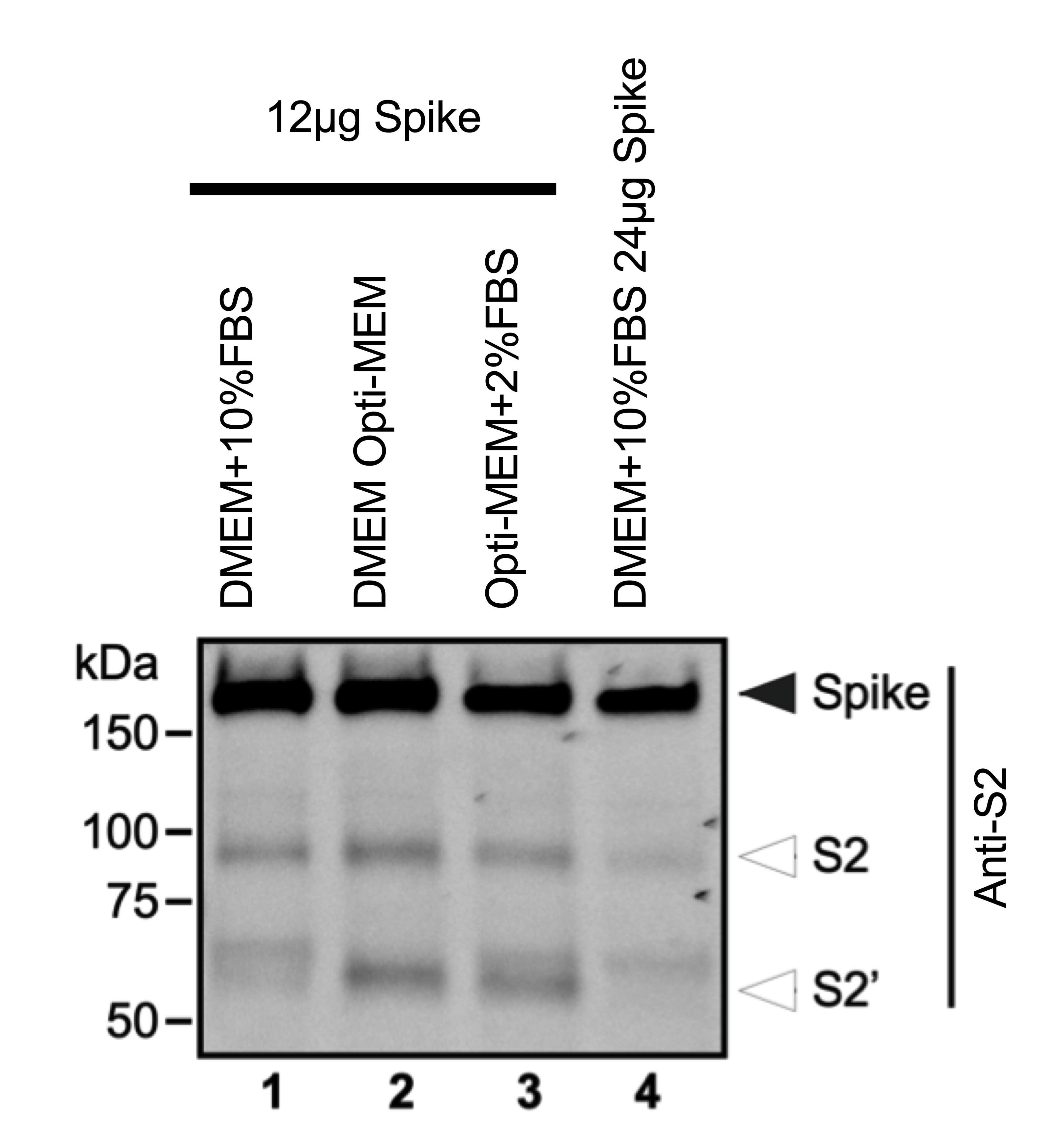

Supplement: FIG S1 [file mbio.02492-20-sf001.tiff]

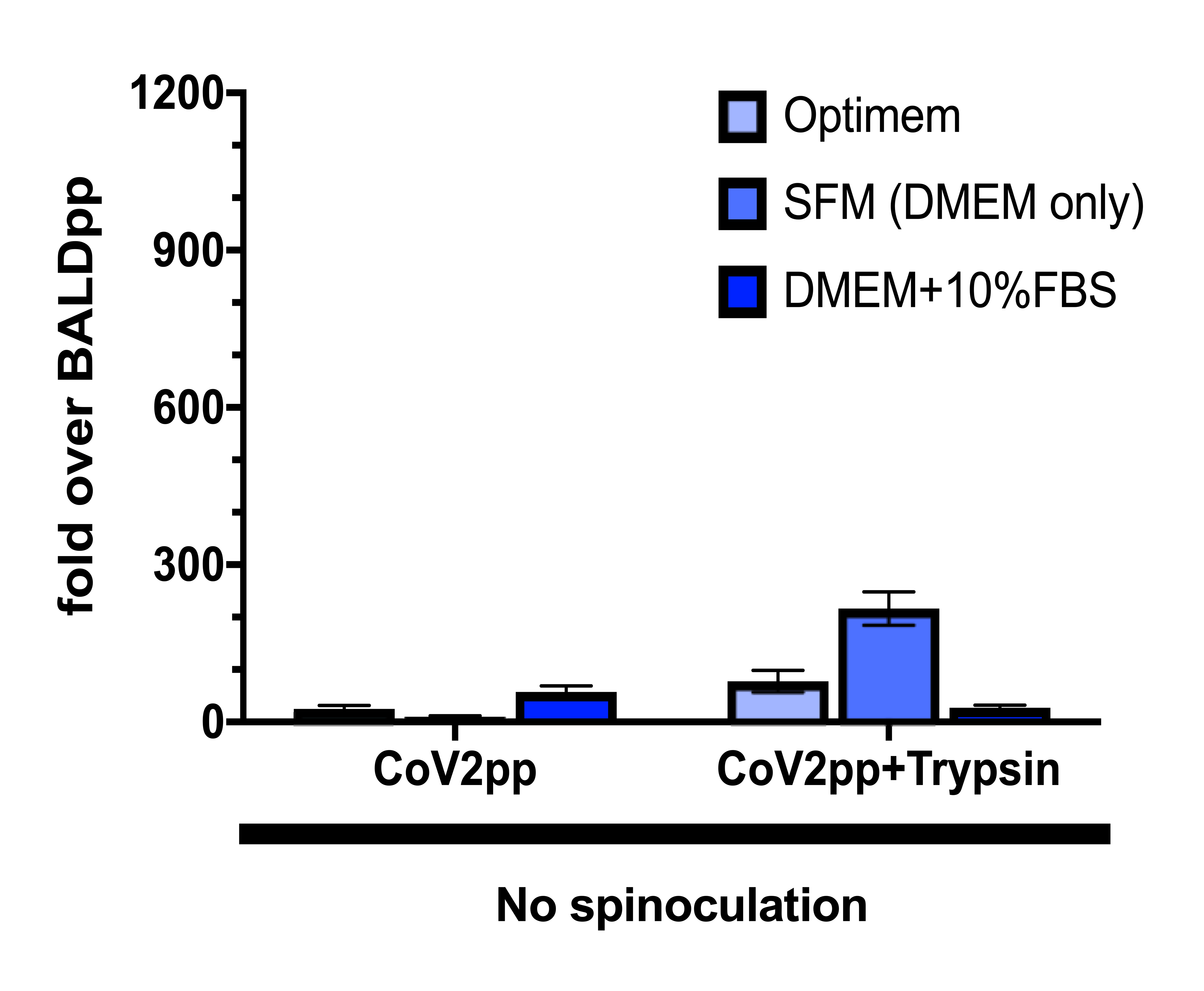

Supplement: FIG S2 [file mbio.02492-20-sf002.tiff]

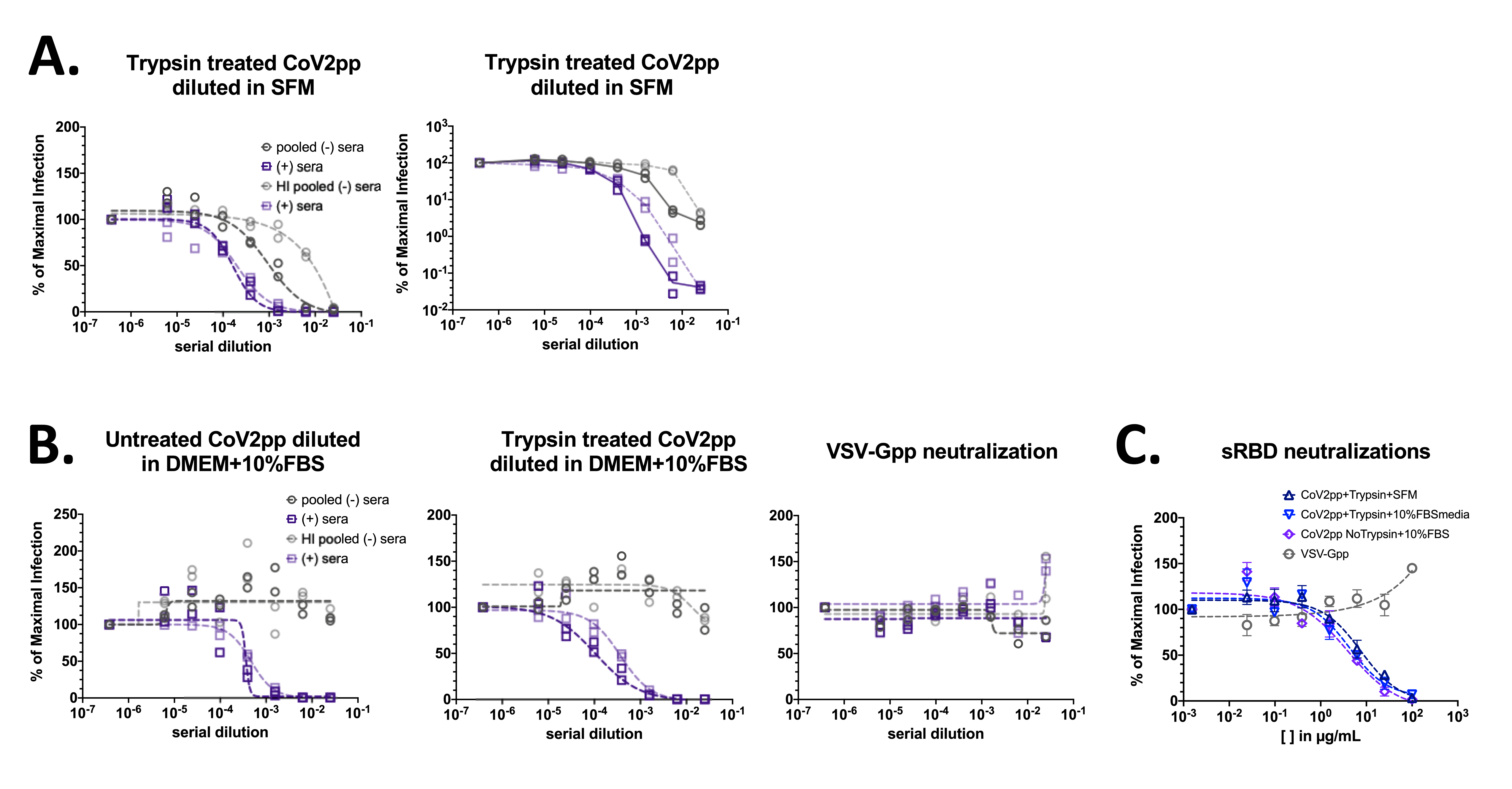

Supplement: FIG S3 [file mbio.02492-20-sf003.tiff]

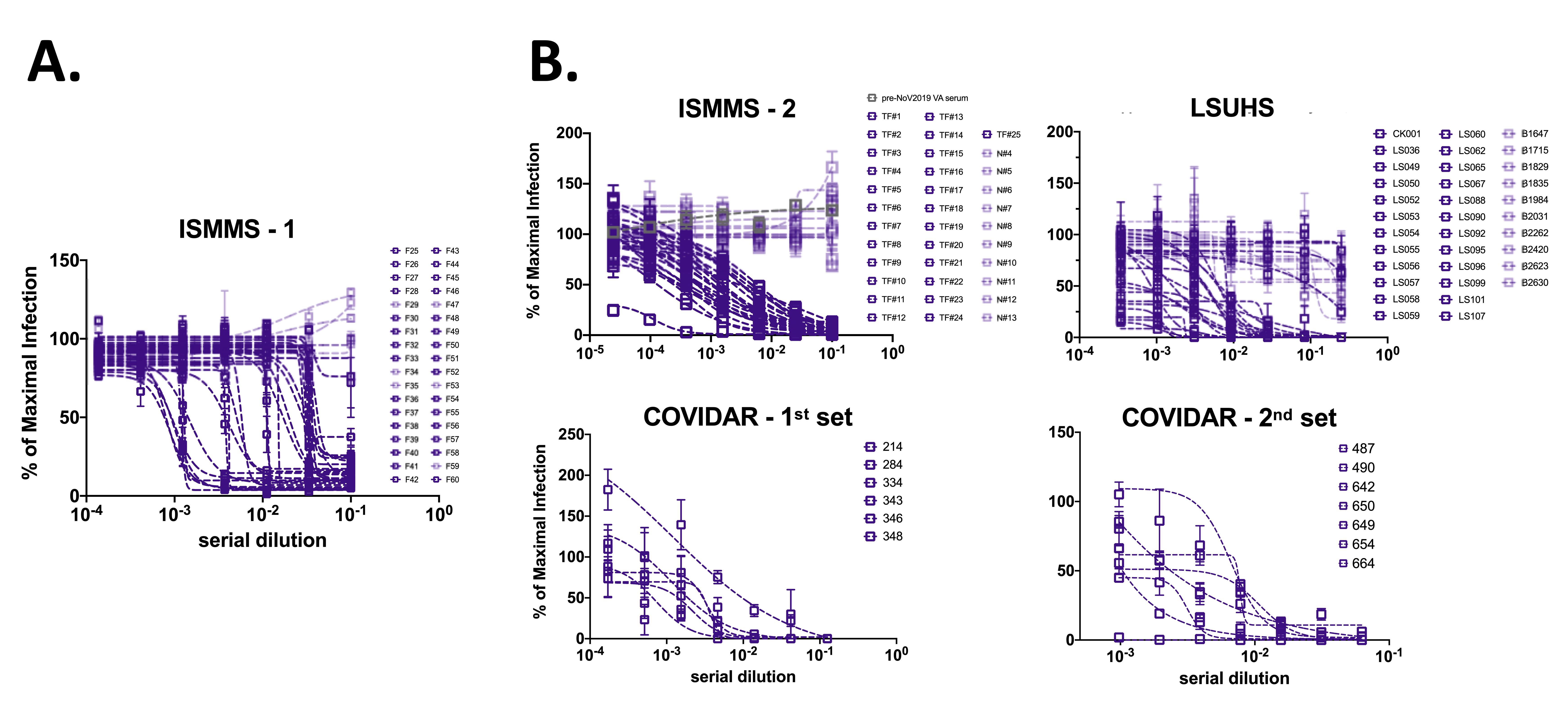

Supplement: FIG S4 [file mbio.02492-20-sf004.tiff]

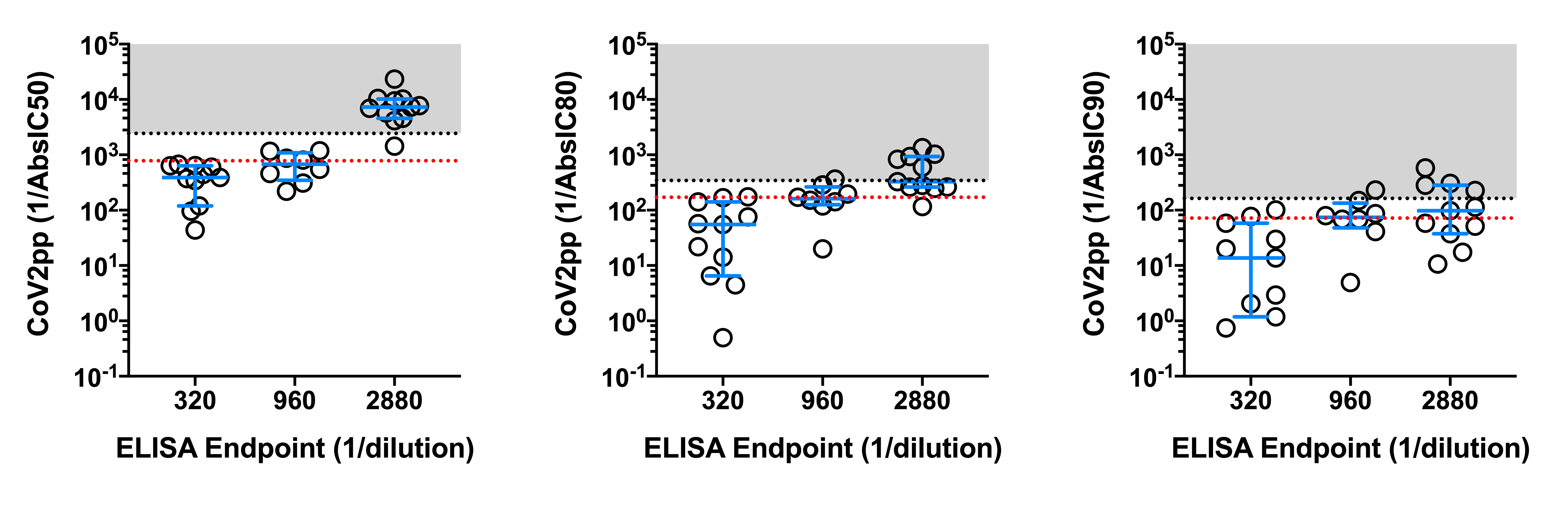

Supplement: FIG S5 [file mbio.02492-20-sf005.tiff]

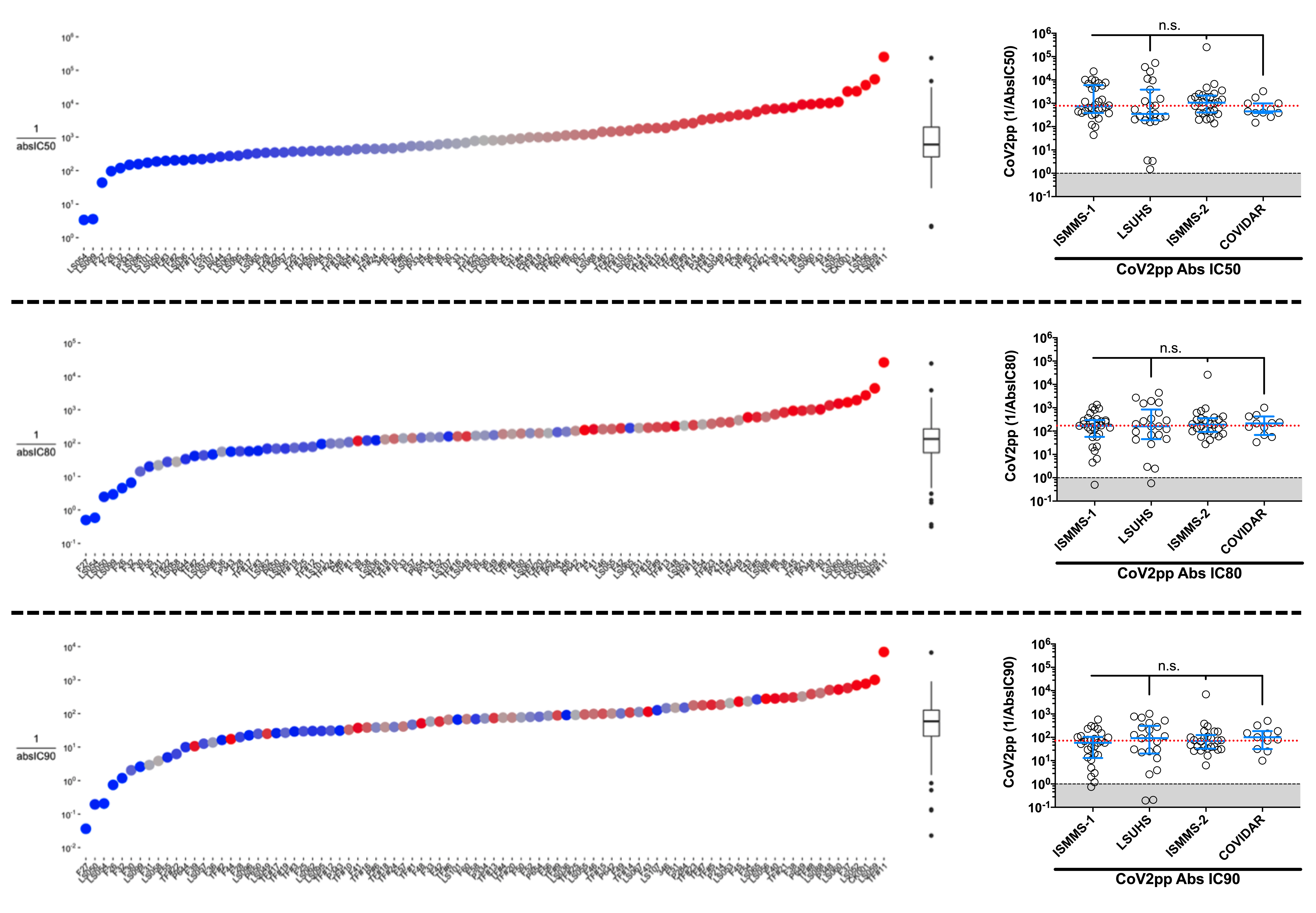

Supplement: FIG S7 [file mbio.02492-20-sf007.tiff]

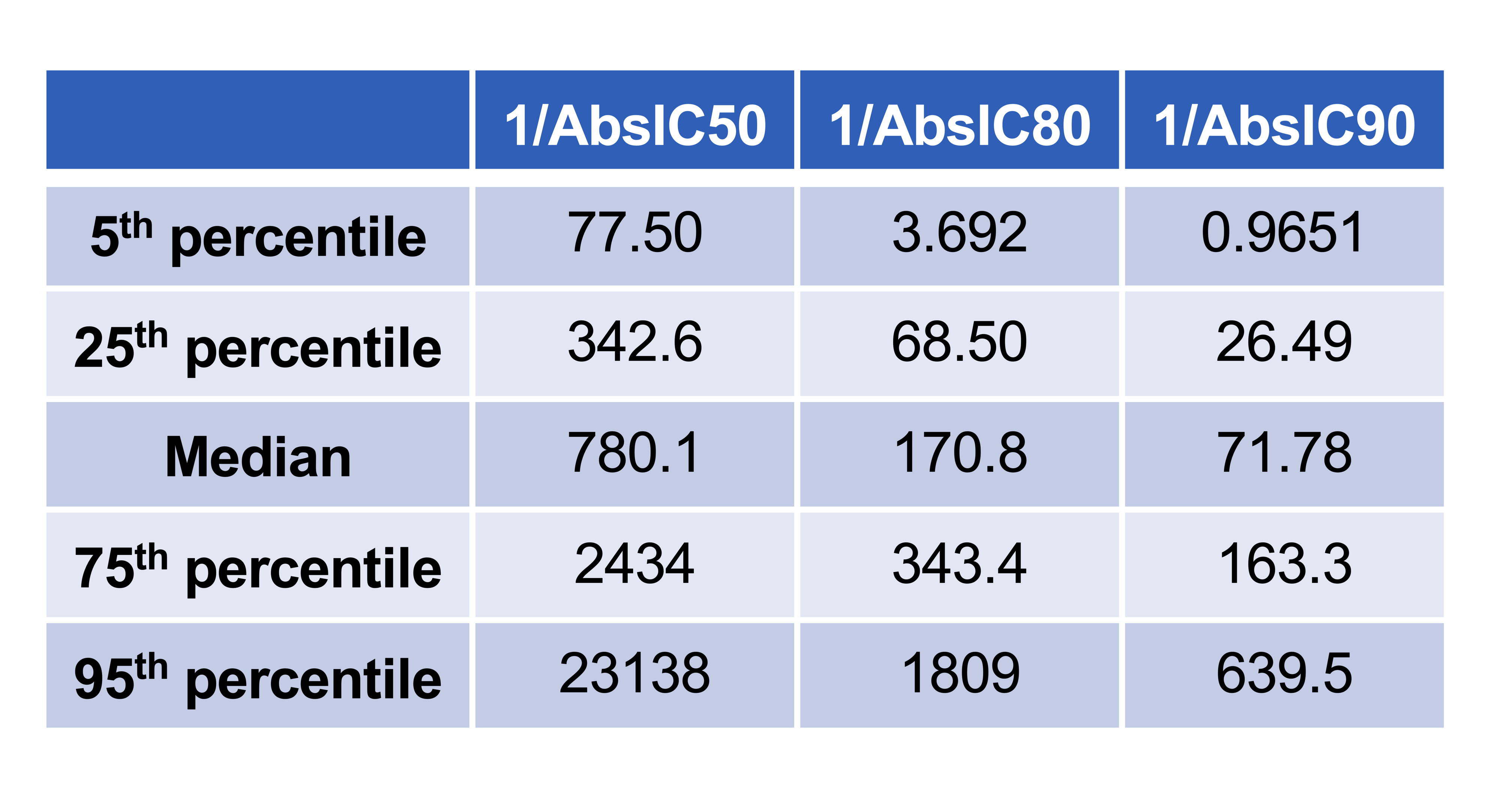

Supplement: TABLE S1 [file mbio.02492-20-st001.tiff]

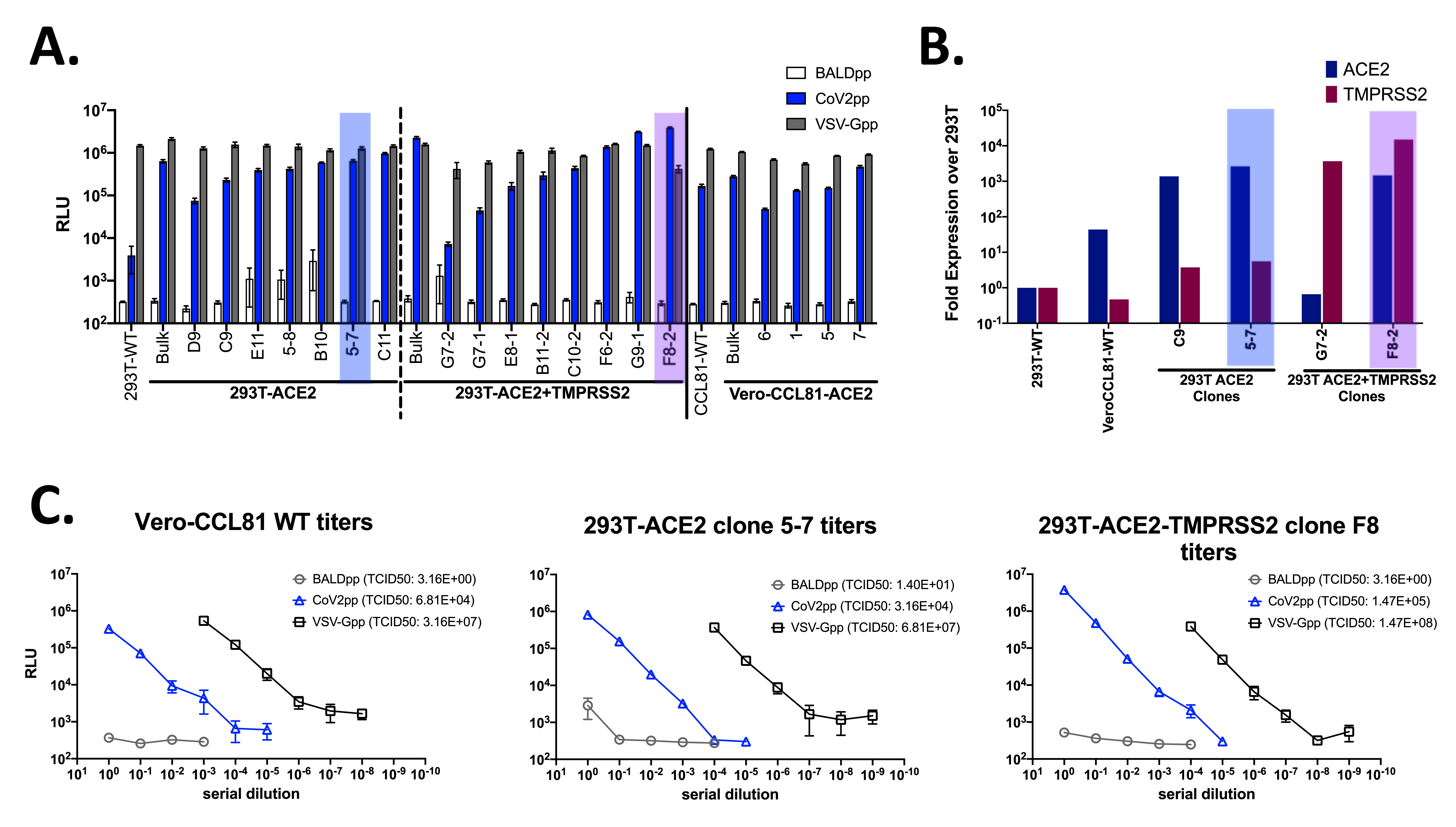

Supplement: FIG S8 [file mbio.02492-20-sf008.tiff]

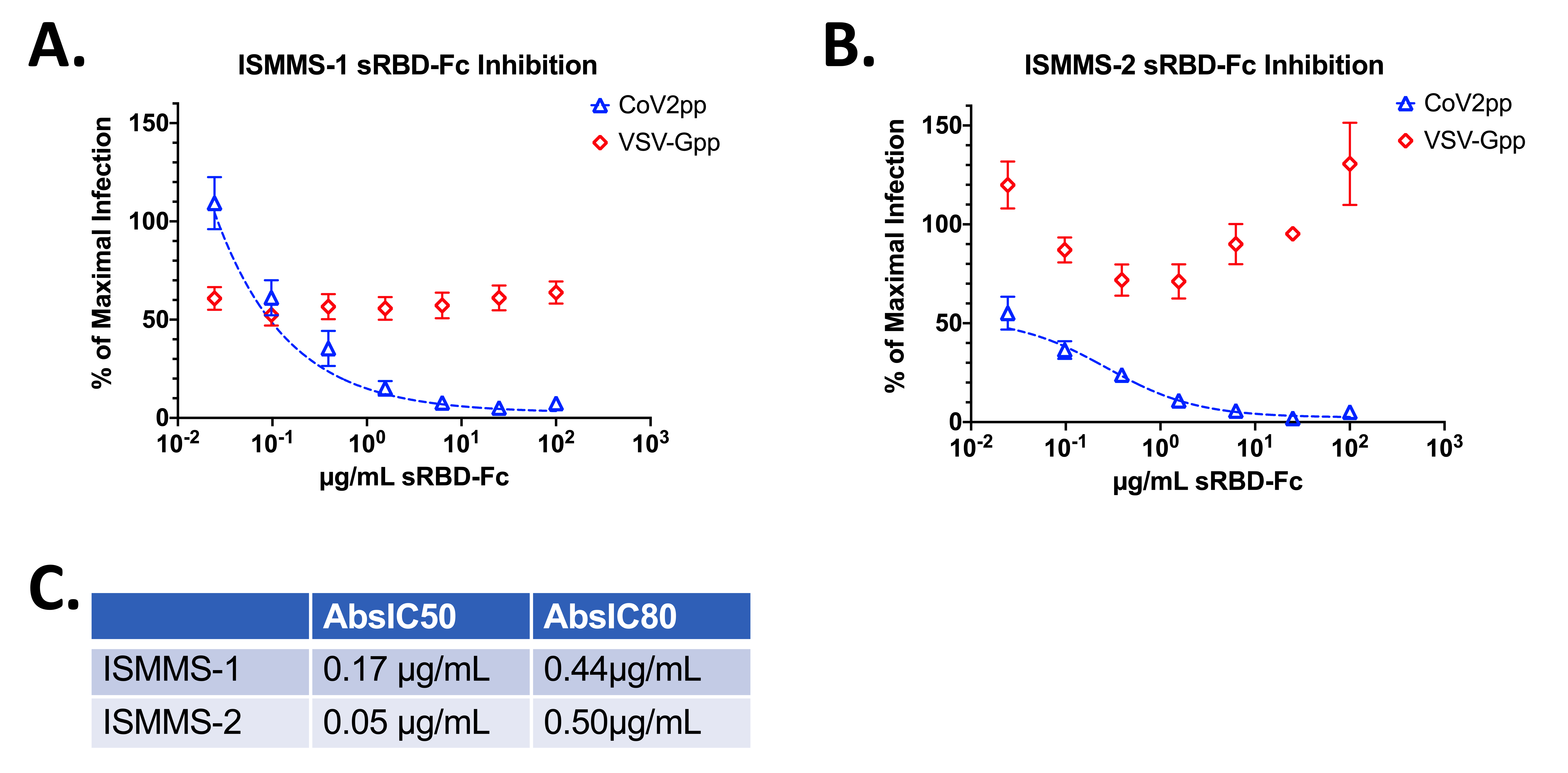

Supplement: FIG S6 [file mbio.02492-20-sf006.tiff]
